# New Cyclic Pentapeptides from the Mangrove-Derived *Aspergillus fumigatus* GXIMD 03099

**DOI:** 10.3390/md22060282

**Published:** 2024-06-16

**Authors:** Yu Wang, Guangping Cao, Yuman Gan, Xiao Lin, Xiangxi Yi, Longyan Zhao, Yonghong Liu, Chenghai Gao, Meng Bai

**Affiliations:** 1 Guangxi Key Laboratory of Marine Drugs, Guangxi University of Chinese Medicine, Nanning 530200, China; 18856101619@163.com (Y.W.); cao15765981927@163.com (G.C.); gan_ym2018@163.com (Y.G.); linxiaolegend@163.com (X.L.); yixiangxi2017@163.com (X.Y.); longyanzhao@gmail.com (L.Z.); yonghongliu@scsio.ac.cn (Y.L.); 2 Institute of Marine Drugs, Guangxi University of Chinese Medicine, Nanning 530200, China

**Keywords:** mangrove-derived fungus, *Aspergillus fumigatus*, cyclic pentapeptides, biological activity

## Abstract

Four new cyclic pentapeptides, avellanins D–G (**1**–**4**), together with four known compounds (**5**–**8**), were isolated from a mangrove-derived *Aspergillus fumigatus* GXIMD 03099 fungus from *Acanthus ilicifolius* L. Their structures were elucidated by analysis of HRESIMS, NMR, and ESI-MS/MS data. Their absolute configurations were determined by X-ray diffraction analysis and Marfey’s method. Compounds **1**–**8** were screened for insecticidal and antibacterial activities. Compound **2** showed insecticidal activity against newly hatched larvae of *Culex quinquefasciatus* with an LC_50_ value of 86.6 µM; compound **4** had weak activity against *Vibrio harveyi* with an MIC value of 5.85 µM.

## 1. Introduction

The secondary metabolites of marine-derived fungi contain a variety of structural types, such as polyketides, terpenoids, alkaloids, and peptides, which are recognized as potential resources for drug discovery [1]. Peptides have been widely used in the clinic, and the structure of cyclic peptides is one of the most special peptides [2,3,4,5]. Cyclic peptides (or cyclopeptides), which originate from natural resources, are cyclic compounds mainly composed of proteinogenic or non-proteinogenic amino acids linked together by amide bonds [6]. According to the composition of amino acid residues in the core ring, the structure of fungal cyclic peptides can be divided into cyclic dipeptides, tripeptides, tetrapeptides, pentapeptides, hexapeptides, heptapeptides, octapeptides, nonapeptides, decapeptides, etc. [7]. Most of the cyclic peptides showed significant biological activities, such as cytotoxicity [8], antitubercular [9], antiviral [10], anti-inflammatory [11], antimicrobial [12], and pancreatic lipase (PL) inhibitory activities [13]. Due to the particularly complex and interesting chemical structures and wide diversity of biological activities of cyclic peptides, they have been attracting significant attention from chemists and pharmacologists [14,15,16].

**Figure 1:**
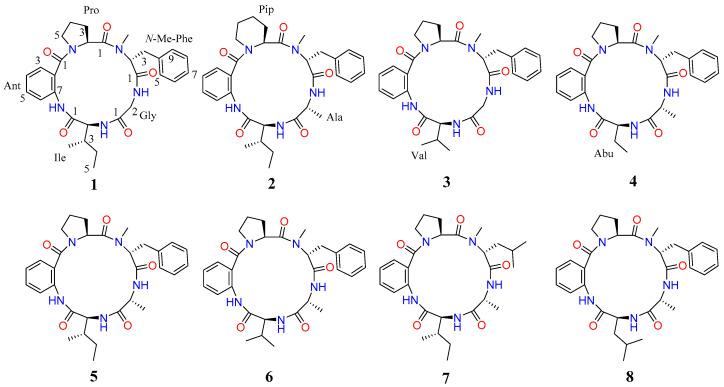
Structures of compounds **1**–**8**.

Cyclic pentapeptides, a subclass of cyclic peptides, are usually constructed by five amino acid residues. To date, about 60 cyclic pentapeptides have been characterized [6,7]. Among them, avellanins A–C were exclusively found in *Hamigera avellanea*, *Hamigera* ingelheimensis, and *Aspergillus petrakii*, respectively. Avellanin A elevates blood pressure, avellanin B improves the activity of antineoplastic drugs, and avellanin C exhibits quorum-sensing inhibitory activity [17,18,19]. During our continuing efforts to identify new bioactive metabolites from mangrove-derived fungi [20,21,22,23], the extracts from the mangrove *Acanthus ilicifolius* L., specifically from fungi *Aspergillus fumigatus* GXIMD 03099, were found to display insecticidal activity and antibacterial activity in preliminary screening. As a result, chemical investigation into the ethyl acetate (EtOAc) extract led to the isolation and identification of eight cyclic peptide compounds, including four new cyclic pentapeptides, avellanins D–G (**1**–**4**) (Figure 1), and four known analogues, avellanin A (**5**) [18], avellanin B (**6**) [18], PF1171E (**7**) [24], and PF1171B (**8**) [24]. Some of the isolated compounds showed significant insecticidal activity and antibacterial activity. Herein, we describe the isolation, structure elucidation, and bioactivities of these compounds.

## 2. Results

### 2.1. Structural Elucidation

In this study, the suitable crystals of compound **5** were obtained from methanol solutions. Analysis of the single-crystal X-ray diffraction data using Cu Kα radiation (with a Flack parameter of 0.06(6)) confirmed the absolute configuration of compound **5**, which was determined to be _L_-Pro-Ant-_L_-Ile-_D_-Ala-*N*-Me-_D_-Phe and named avellanin A.

Compound **1** was obtained as yellow amorphous powder, and its molecular formula was C30H37N5O5 on the basis of HRESIMS ion at *m*/*z* 548.2864 [M + H]^+^ (calcd for 548.2873), implying 15 degrees of unsaturation. Careful analysis of the 1H NMR data of compound **1** (Table 1) displayed the signals for three amide protons (*δ*_H_ 9.62 (s), 7.78 (dd, *J* = 7.5, 4.0 Hz), and 7.20 (d, *J* = 9.0 Hz)), one N-methyl group (*δ*_H_ 3.00 (s)), and six α-amino protons (*δ*_H_ 5.30 (dd, *J* = 12.0, 4.5 Hz), 4.38 (dd, *J* = 8.5, 4.5 Hz), 4.26 (dd, *J* = 17.0, 8.0 Hz), 3.66 (dd, *J* = 9.5, 3.5 Hz), 3.63 (m), and 3.29 (m)). Additionally, the 13C NMR and DEPT135 data of compound **1** (Table 1) exhibited thirty carbon signals, including five carbonyl carbons (*δ*_C_ 173.5, 169.9, 169.4, 168.9, and 167.4), twelve aromatic carbons (*δ*_C_ 137.7, 135.7, 130.9, 128.3 × 4, 127.5, 126.4, 123.9, 123.2, and 120.0), six methines (*δ*_C_ 50.1, 42.6, 32.5, 28.1, 24.6, and 24.1), four methylenes (*δ*_C_ 58.8, 58.1, 56.7, and 36.2), and three methyls (*δ*_C_ 31.6, 15.7, and 11.7). Detailed analysis of these aforementioned data indicated that compound **1** was a peptide with five amino acid residues. These signals accounted for 14 of 15 degrees of unsaturation; to satisfy the remaining degree of unsaturation, compound **1** must be cyclic.

**Figure 2:**
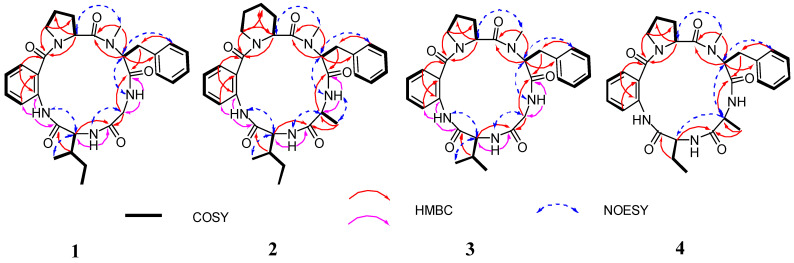
Key ^1^H-^1^H COSY and HMBC correlations for compounds **1**–**4**.

**Figure 3:**
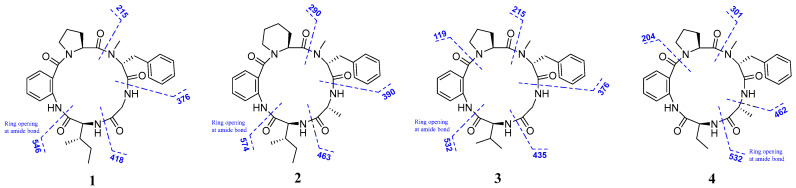
ESI-MS/MS analysis of compounds **1**–**4**.

Furthermore, detailed analysis of the heteronuclear single quantum correlation (HSQC), correlation spectroscopy (COSY), heteronuclear multiple bond correlation (HMBC), and nuclear Overhauser effect spectroscopy (NOESY) spectrum confirmed the cyclic pentapeptides structure of compound **1** (Figure 2) and revealed the presence of proline (Pro), anthranilic acid (Ant), isoleucine (Ile), glycine (Gly), and *N-*methyl-phenylalanine (*N-*Me-Phe). The first fragment of Pro was established by interpretation of COSY correlations from H-2 (*δ*_H_ 4.88) to H-3 (*δ*_H_ 2.11 and 1.39), H-4 (*δ*_H_ 1.85), and H-5 (*δ*_H_ 3.63 and 3.29), and coupled with HMBC correlations from H-2 (*δ*_H_ 4.88) to C-1 (*δ*_C_ 173.5), C-4 (*δ*_C_ 24.6), and Ant-CO (*δ*_C_ 167.4), and from H-5 (*δ*_H_ 3.63 and 3.29) to C-3 (*δ*_C_ 28.1) and Ant-CO (*δ*_C_ 167.4). The Ant residue was identified by COSY correlations from H-3 (*δ*_H_ 7.52) to H-4 (*δ*_H_ 7.16), H-5 (*δ*_H_ 7.47), and H-6 (*δ*_H_ 8.38), and by observations of HMBC correlations from H-3 (*δ*_H_ 7.52) to C-1 (*δ*_C_ 167.4) and C-7 (*δ*_C_ 123.9), and from H-6 (*δ*_H_ 8.38) to C-2 (*δ*_C_ 135.7). For the Ile residues, the presence of COSY correlations from H-2 (*δ*_H_ 4.38) to H-3 (*δ*_H_ 2.01), H-4 (*δ*_H_ 1.37 and 1.20), and H-5 (*δ*_H_ 0.83), and from H-3′ (*δ*_H_ 0.86) to H-3 (*δ*_H_ 2.01), and by observations of HMBC correlations from H-2 (*δ*_H_ 4.38) to C-1 (*δ*_C_ 168.9) and Gly-CO (*δ*_C_ 169.4). Moreover, the Gly residue was identified by COSY correlations from H-2 (*δ*_H_ 4.26 and 3.66) to NH (*δ*_H_ 7.78), and the analysis of HMBC correlations H-2 (*δ*_H_ 4.26 and 3.66) to C-1 (*δ*_C_ 169.4) and *N*-Me-Phe-CO (*δ*_C_ 169.9). The *N*-Me-Phe residue was also established by the COSY correlations from H-2 (*δ*_H_ 5.30) to H-3 (*δ*_H_ 3.40 and 3.10), and from H-5/9 (*δ*_H_ 7.25) to H-6/8 (*δ*_H_ 7.31) and H-7 (*δ*_H_ 7.22), and the observation of HMBC correlations from H-2 (*δ*_H_ 5.30) to C-1 (*δ*_C_ 169.9), C-4 (*δ*_C_ 137.7), and Pro-CO (*δ*_C_ 173.5), as well as from the *N*-methyl protons (*N*-Me, *δ*_H_ 3.00) to C-2 (*δ*_C_ 58.8) and Pro-CO (*δ*_C_ 173.5). Lastly, HMBC correlations from Ant-NH (*δ*_H_ 9.62) to Ile-CO (*δ*_C_ 168.9), from Ile-NH (*δ*_H_ 7.20) to Gly-CO (*δ*_C_ 169.4), from Gly-NH (*δ*_H_ 7.78) to *N*-Me-Phe-CO (*δ*_C_ 169.9), from *N*-Me-Phe-H-2 (*δ*_H_ 5.30) to Pro-CO (*δ*_C_ 173.5), and from Pro-H-2 (*δ*_H_ 4.88) to Ant-CO (*δ*_C_ 167.4) resulted in the sequence of five amino acids. Moreover, the key NOESY correlations between Pro-H-2 (*δ*_H_ 4.88) and Phe-*N*-Me (*δ*_H_ 3.00), Phe-H-2 (*δ*_H_ 5.30) and Gly-NH (*δ*_H_ 7.78), Gly-H-2 (*δ*_H_ 4.26 and 3.66) and Ile-NH (*δ*_H_ 7.20), Ile-H-2 (*δ*_H_ 4.38) and Ant-NH (*δ*_H_ 9.62) established the partial sequence of amino acids, and the ESI-MS/MS fragment ions at *m*/*z* 418 (loss of Ile), *m*/*z* 376 (loss of Ile-Gly), and *m*/*z* 215 (loss of Ile-Gly-*N*-Me-Phe) (Figure 3 and Figure S10) also established the sequence order of amino acid residues. Therefore, the planar structure of compound **1** was Pro-Ant-Ile-Gly-*N*-Me-Phe (Figure 1).

**Figure 4:**
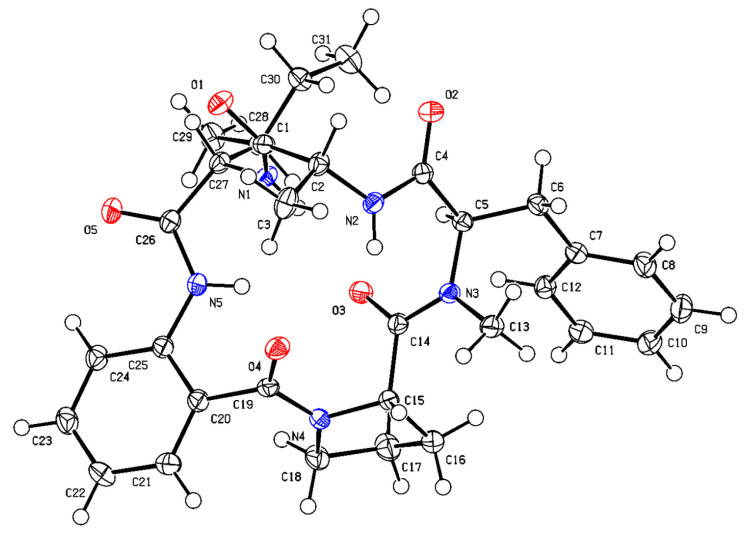
Single-crystal X-ray structure of compound **5**.

The absolute configuration of the amino acid residues of compound **1** was established by HPLC-MS analysis of their acid hydrolysates derivatized with Marfey’s method [25]. The L-Pro and L-Ile in compound **1** were confirmed by analyzing the retention time of their derivatives and standards on HPLC-MS (Figure S11). However, the absolute configuration of the *N*-Me-Phe could not be identified by Marfey’s method, as standard amino acids were not available. Compound **1** displayed the same *N*-Me-Phe amino acid residues as observed in avellanin A (**5**) [18]. Fortunately, we obtained the crystals of compound **5** in MeOH/H_2_O, with a Flack parameter of 0.06(6) (Figure 4). Additionally, the absolute configuration of the *N*-Me-Phe of compound **1** may be consistent with that of compound **5** by their common biosynthetic pathway. Finally, the *N*-Me-Phe of compound **1** was identified as *N*-Me-_D_-Phe by comparing the retention time of the *N*-Me-Phe of compounds **1** and **5** with Marfey’s method (Figure S11). Finally, the structure of compound **1** was elucidated as _L_-Pro-Ant-_L_-Ile-Gly-*N*-Me-_D_-Phe and named avellanin D.

Compound **2** was obtained as yellow powder. Its HRESIMS spectrum showed the ion at *m*/*z* 598.3001 [M + Na]^+^ (calcd for 598.3005), suggesting its molecular formula as C_32_H_41_N_5_O_5_ with 15 degrees of unsaturation. The ^1^H and ^13^C NMR data (Table 1) of compound **2** were similar to those of compound **5**, the only obvious difference being the additional presence of one methylene (*δ*_H_ 1.49 and 1.30, and *δ*_C_ 17.6) in compound **2**. Detailed analysis of the NMR spectra indicated that the Pro residue in compound **5** was replaced by a Pip unit in compound **2**. The results were confirmed by the COSY correlations of H-2 (*δ*_H_ 4.86) to H-3 (*δ*_H_ 1.69 and 1.34), H-4 (*δ*_H_ 1.49 and 1.30), H-5 (*δ*_H_ 1.62), and H-6 (*δ*_H_ 3.57 and 3.40), and HMBC correlations of H-2 (*δ*_H_ 4.86) to C-1 (*δ*_C_ 173.7), C-4 (*δ*_C_ 17.6), and Ant-CO (*δ*_C_ 171.9), and H-6 (*δ*_H_ 3.57 and 3.40) to C-4 (*δ*_C_ 17.6) and Ant-CO (*δ*_C_ 171.9) (Figure 2). Therefore, the amino acid sequence of compound **2** was assigned as Pip-Ant-Ile-Ala-*N*-Me-Phe by the key 2D NMR correlations (Figure 2) and further supported by ESI-MS/MS analysis (Figure 3 and Figure S21). The absolute configurations of the amino acid residues of compound **2** were identified using Marfey’s method (Figure S22) [25]. In addition, basis on the absolute configuration of avellanins A and B (**5** and **6**) [18], and a shared biogenesis in the same fungus. Thus, the completed structure of compound **2** was elucidated as _L_-Pip-Ant-_L_-Ile-_D_-Ala-*N*-Me-_D_-Phe and named avellanin E.

Compound **3** was obtained as a yellow amorphous powder. It is assigned the molecular formula C_29_H_35_N_5_O_5_ on the basis of HRESIMS spectrum, which revealed 15 degrees of unsaturation. Carefully analysis of the ^1^H and ^13^C NMR data (Table 2) of compound **3** closely resemble those of compound **1**, indicating that compound **3** was an analogue of compound **1**, the main difference being one amino acid constitution, corresponding to the loss of a methylene group from compound **1**. The above results were confirmed by the COSY correlations of H-4 (*δ*_H_ 0.88) and H-5 (*δ*_H_ 0.86) to H-3 (*δ*_H_ 2.31) and H-2 (*δ*_H_ 5.29), and HMBC correlations of H-3 (*δ*_H_ 2.31) to C-1 (*δ*_C_ 168.9) and C-2 (*δ*_C_ 58.7), and H-2 (*δ*_H_ 5.29) to C-1 (*δ*_C_ 168.9), C-3 (*δ*_C_ 29.4), C-4 (*δ*_C_ 19.0), C-5 (*δ*_C_ 16.9), and Gly-CO (*δ*_C_ 169.4) (Figure 2). Indeed, analysis of 2D NMR data established the amino acid sequence of compound **3** to be Pro-Ant-Val-Gly-*N*-Me-Phe. The ESI-MS/MS data also supported the sequence order of amino acid residues (Figure 3 and Figure S32). In addition, the absolute configuration of compound **3** was identified using Marfey’s method (Figure S33) [25]. Thus, the completed structure of compound **3** was elucidated as _L_-Pro-Ant-_L_-Val-Gly-*N*-Me-_D_-Phe and named avellanin F.

Compound **4** was obtained as a white amorphous powder, and its molecular formula C_29_H_35_N_5_O_5_ was established from HRESIMS, which revealed 15 degrees of unsaturation. The ^1^H and ^13^C NMR spectra (Table 2) of compound **4** were similar to those of compound **5**, except for the absence of one methine group and one methyl group of Ile in compound **5**, corresponding to a mass difference of 28 amu compared to compound **5**. The COSY spectrum indicated a proton spin system, H-2 (*δ*_H_ 4.42), H-3 (*δ*_H_ 2.13 and 1.73), and H-4 (*δ*_H_ 1.00), and HMBC correlations of H-2 (*δ*_H_ 4.42) to C-1 (*δ*_C_ 171.7), C-3 (*δ*_C_ 25.1), C-4 (*δ*_C_ 11.1), and Ala-CO (*δ*_C_ 174.8), H-3 (*δ*_H_ 2.13 and 1.73) to C-1 (*δ*_C_ 171.7), C-2 (*δ*_C_ 57.6), and C-4 (*δ*_C_ 11.1), and H-4 (*δ*_H_ 1.00) to C-2 (*δ*_C_ 57.6) and C-3 (*δ*_C_ 25.1) (Figure 2). The interpretation of the HMBC spectrum of compound **4** clarified the sequence of amino acid to be Pro-Ant-Abu-Ala-*N*-Me-Phe, which was further confirmed by the NOESY correlations and EIS-MS/MS fragmentation (Figure 2, Figure 3 and Figure S43). According to Marfey’s method (Figure S44) [25], the absolute configuration of compound **4** was identified, and the locations of _L_-Pro, _L_-Abu, _D_-Ala, and *N*-Me-_D_-Phe were assigned. Finally, the completed structure of compound **4** was elucidated as _L_-Pro-Ant-_L_-Abu-_D_-Ala-*N*-Me-_D_-Phe and named avellanin F.

Four known compounds were isolated and identified as avellanin A (**5**) [18], avellanin B (**6**) [18], PF1171E (**7**) [24], and PF1171B (**8**) [24] by comparing their physical and spectroscopic data with those reported in the literature.

### 2.2. Biological Activity

Compounds **1**–**8** were subjected to several biological assays available in our laboratories, including insecticidal and antibacterial activity tests. The result showed that compound **2** exhibited insecticidal activity against newly hatched larvae of *Culex quinquefasciatus* with an LC_50_ value of 86.6 µM, and compound **4** had weak activity against *Vibrio harveyi* with an MIC value of 5.85 µM.

## 3. Materials and Methods

### 3.1. General Experimental Procedures

Optical rotations were determined using an InsMark digi300 spirometer (InsMark, Shanghai, China). CD spectra were measured with a JASCO J-1500 digital polarimeter (JASCO, Easton, PA, Tokyo, Japan). IR spectra were recorded using a PerkinElmer DTGS FT/IR-L1600400 (using KBr disks, PerkinElmer, Waltham, MA, USA) spectrophotometer. One-dimensional and two-dimensional NMR spectra were measured on a Bruker AV-400 MHz spectrometer and AV-500 MHz spectrometer, respectively, with TMS as the internal standard (Bruker, Fällanden, Switzerland). Semi-preparative HPLC was performed on a SHIMADZU LC-2030C series (Shimadzu, Kyoto, Japan) with a DAD detector using a COSMOSIL 5C_18_-MS-II column (10 × 250 mm, 7 µm, Cosmosil, Kyoto, Japan). Melting points were determined on an SGW X-4B micromelting point apparatus and were uncorrected (SHYDWG, Shanghai, China). HR-ESI-MS spectra were obtained on a Bruker Daltonics Apex-Ultra 7.0 T spectrometer (Bruker Corporation, Billerica, MA, USA) and a WATERS Xevo G2-S Qtof Quadrupole Timeof-Flight Mass Spectrometry (Waters, Milford, MA, USA).

### 3.2. Fungal Material, Isolation, and Purification

The fungal strain *Aspergillus fumigatus* GXIMD 03099 was isolated from the mangrove *Acanthus ilicifolius* L., collected in the Guangxi Shankou Mangrove Nature Reserve in July, 2020, and was identified according to its morphological characteristics and a molecular biological protocol by 18S rRNA amplification and sequencing of the ITS region. The sequence data have been submitted to GenBank, with accession number ON668102. The fungal strain was cultivated in rice solid-substrate medium (100 Erlenmeyer flasks, each containing 80 g of rice and 0.5 g of sea salt in 100 mL of distilled H_2_O in 1 L Erlenmeyer flasks) at room temperature under static conditions and daylight for 30 days.

The solid rice medium was extracted with EtOAc. The organic extract was concentrated in vacuo to yield an oily residue (76.5 g), which was subjected to silica gel column chromatography (CC) using petroleum ether/EtOAc (*v*/*v*, gradient 100:0–0:100) and EtOAc/MeOH (*v*/*v*, gradient 100:0–0:100) to generate ten fractions (Fr. 1–Fr. 10).

Fr. 5 (9.45 g) was separated by silica gel CC, eluted with petroleum ether–EtOAc (from 10:0 to 0:10) and EtOAc–MeOH (from 3:1 to 0:10) to afford eight subfractions (5a–5h). Subfraction 5e was further separated by semi-preparative HPLC (MeOH/H_2_O, 75:25, *v*/*v*) to obtain compounds **2** (2.88 mg, *t*_R_ = 44.92 min), **7** (2.96 mg, *t*_R_ = 20.12 min) and **8** (3.52 mg *t*_R_ = 30.45 min). Subfraction 5f was further separated by semi-preparative HPLC (MeOH/H_2_O, 80:20, *v*/*v*) to obtain compounds **4** (2.52 mg, *t*_R_ = 6.43 min) and **6** (12.86 mg, *t*_R_ = 39.56 min).

Fr. 7 (10.29 g) was separated by silica gel CC, eluted with petroleum ether–EtOAc (from 10:0 to 0:10) and EtOAc–MeOH (from 3:1 to 0:10) to afford nine subfractions (7a–7i). Subfraction 7b was isolated by CC on silica gel eluting petroleum ether–EtOAc (from 10:0 to 0:10) and EtOAc–MeOH (from 3:1 to 0:10) to afford thirteen subfractions (7b-a–7b-m), and subfraction 7b-i was further separated by semi-preparative HPLC (MeOH/H_2_O, 80:20, *v*/*v*) to obtain compounds **3** (5.42 mg, *t*_R_ = 18.57 min) and **1** (14.89 mg, *t*_R_ = 44.92 min).

Fr. 8 (10.1 g) was separated by silica gel CC, eluted with petroleum ether–EtOAc (from 10:0 to 0:10) and EtOAc–MeOH (from 3:1 to 0:10) to afford twelve subfractions (8a–8l). Subfraction 8f was further separated by semi-preparative HPLC (MeOH/H_2_O, 95:5, *v*/*v*) to obtain compound **5** (6.82 mg, *t*_R_ = 22.50 min).

Avellanin D (**1**): yellow powder. [α${]}_{D}^{24}$ +12.0 (*c* 0.1, MeOH); UV (MeOH) λ_max_ (log ε) 285, 251, 204 nm; CD (c 2 × 10^−4^ mol/L, MeOH) λ_max_ (Δε) 200 (−7.5), 231 (17.3) nm; IR (KBr) ν_max_ 3349, 2963, 1684, 1524, 1419 cm^−1^; ^1^H and ^13^C NMR data, see Table 1; HR-ESI-MS *m*/*z* 548.2864 [M + H]^+^ (calcd. for C_30_H_38_N_5_O_5_, 548.2873).

Avellanin E (**2**): yellow powder. [α${]}_{D}^{24}$ −10.0 (*c* 0.05, MeOH); UV (MeOH) λ_max_ (log ε) 287, 246, 208 nm; CD (c 2 × 10^−4^ mol/L, MeOH) λ_max_ (Δε) 205 (−22.7), 230 (37.6) nm; IR (KBr) ν_max_ 3365, 2969, 1621, 1592, 1452 cm^−1^; ^1^H and ^13^C NMR data, see Table 1; HR-ESI-MS *m*/*z* 598.3001 [M + Na]^+^ (calcd. for C_32_H_41_N_5_O_5_Na, 598.3005).

Avellanin F (**3**): yellow powder. [α${]}_{D}^{24}$ +16.0 (*c* 0.1, MeOH); UV (MeOH) λ_max_ (log ε) 287, 250, 203 nm; CD (c 2 × 10^−4^ mol/L, MeOH) λ_max_ (Δε) 237 (18.3), 291 (−2.4) nm; IR (KBr) ν_max_ 3363, 2919, 1617, 1523, 1419, cm^−1^; ^1^H and ^13^C NMR data, see Table 2; HR-ESI-MS *m*/*z* 534.2696 [M + H]^+^ (calcd. for C_29_H_36_N_5_O_5_, 534.2716).

Avellanin G (**4**): white powder. [*α*${]}_{D}^{24}$ +34.0 (*c* 0.1, MeOH); UV (MeOH) *λ*_max_ (log *ε*) 290, 250, 201 nm; CD (*c* 2 × 10^−4^ mol/L, MeOH) *λ*_max_ (Δ*ε*) 200 (−6.1), 232 (11.6) nm; IR (KBr) *ν*_max_ 3471, 2925, 1649, 1599, 1422 cm^−1^; ^1^H and ^13^C NMR data, see Table 2; HR-ESI-MS *m*/*z* 534.2706 [M + H]^+^ (calcd. for C_29_H_36_N_5_O_5_, 534.2716).

### 3.3. X-ray Crystallographic Analyses of Compound ***5***

Colorless crystals of compound **5** were obtained from MeOH/H_2_O. Single-crystal X-ray diffraction data were collected on an *Xcalibur*, *Atlas*, *Gemini ultra*-diffractometer with Cu K*α* radiation (*λ* = 1.54184 Å) at 99.9 (3) K, respectively. The structure was solved by direct methods (ShelXT) and refined with the ShelXL refinement package using least squares minimization. All non-hydrogen atoms were refined anisotropically, and all hydrogen atoms were placed in idealized positions and refined relatively isotropically with a riding model. Crystallographic data of compound **5** have been deposited in the Cambridge Crystallographic Data Centre with deposition number CCDC 2323898. Copies of the data can be obtained, free of charge, on application to the Director, CCDC, 12 Union Road, Cambridge CB21EZ, UK (fax: +44-(0)1223-336033, or E-mail: deposit@ccdc.cam.ac.uk).

Crystal data for compound **5**: C_31_H_39_N_5_O_5_·H_2_O, mp. 117–119 °C, M_r_ = 561.67, monoclinic, a = 7.82520 (10) Å, b = 18.63970 (10) Å, c = 10.36950 (10) Å, *α* = 90°, *β* = 107.4970 (10) °, *γ* = 90°, V = 1442.51 (3) Å^3^, space group P2_1_, Z = 2, D_x_ = 1.293 g/cm^3^, *μ* (Cu Kα) = 0.721 mm^−1^, and F (000) = 600.0. Independent reflections: 5709 (R_int_ = 0.0242). The final R_1_ value was 0.0274, wR_2_ = 0.0713 (I >2σ (I)). Flack parameter = 0.06(6).

### 3.4. Acid Hydrolysis and Marfey’s Analysis Methods

Hydrolysis and Marfey’s analysis were carried out according to Xiao Lin et al. [25]. Compounds **1**–**4** (100 μg each) were dissolved in 100 μL of 6 M HCl and hydrolyzed in a pressure-resistant reaction flask at 110 °C for 24 h, then the HCl was removed by evaporation under a stream of N_2_ gas. The hydrolysis product was dissolved in 50 μL of 1 M NaHCO_3_ to adjust the pH to 7–8 and reacted with 10 μL of Marfey’s reagent (1-fluoro-2, 4-dinitrophenyl-5-_L_-leucinamide, _L_-FDLA; 1% solution in acetone) at 40 °C for 1 h, and then 50 μL of 1 M HCl was used to neutralize the reactant pH to 2–3. Finally, the mixture was diluted with MeCN (800 μL) and filtered. Amino acid standards _L_-Pip, _L_-Pro, _L_-Val, _L_-Ile, and _L_-Abu were derivatized with _L_-FDLA. The _L_-Pro, _L_-Ile, _D_-Ala, and N-Me-_D_-Phe from compound **5** crystals were used as standards for comparison with the hydrolysate derivatives of compounds **1**–**4**. Marfey’s derivatives of the compounds and the L-FDAA standards described above were analyzed using HPLC-DAD-MS in positive ion mode using a WATERS Xevo G2-S Qtof Quadrupole Timeof-Flight Mass Spectrometry (Waters, Milford, MA, USA).

### 3.5. Insecticidal Activities

The insecticidal activity against *Culex quinquefasciatus* larvae was evaluated according to methods reported in the literature [21]. Newly hatched larvae were housed at 25 ± 1 °C and 80% relative humidity. Two sets of replicates were set up with dimethyl sulfoxide as a negative control, azadirachtin as a positive control, and artificial feed as a blank control. The number of dead larvae was recorded on 2nd, 4th, 6th, and 8th days after treatment.

### 3.6. Antibacterial Activities

Antibacterial activity was determined against nine pathogenic bacteria [26], including six Gram-positive bacteria—*Methicillin-resistant Staphylococcus aureus* (ATCC 43300), *Candida albicans* (ATCC 10231), *Staphylococcus albus* (ATCC 8799), *Staphylococcus aureus* (ATCC 6538), *Bacillus subtilis* (ATCC 21332), and *Xanthomonas Campestris* (ATCC 33913) —and three Gram-negative bacteria—*Vibrio parahaemolyticus* (ATCC 17802), *V. alginolyticus* (ATCC 17749), and *V. harveyi* (ATCC 14126)—by the microplate assay method. Chloromycetin was used as the positive control.

## 4. Conclusions

In conclusion, we reported four new cyclic pentapeptides named avellanins D–G (**1**–**4**). The significant difference between compounds **1**–**4** is that compound **2** contains an uncommon Pip amino acid. The biological activity screening showed that only compounds **2** and **4** had insecticidal and anti-*Vibrio* activity, respectively. Therefore, the presence of Pip and Abu amino acid residues appears to be crucial for insecticidal and anti-*Vibrio* activity.

## Figures and Tables

**Table 1:** ^1^H NMR and ^13^C NMR data for compounds **1**–**2**.

	1 ^a^				2 ^b^		
Unit	Position	*δ*_H_, (*J* in Hz)	*δ*_C_, Type	Unit	Position	*δ*_H_, (*J* in Hz)	*δ*_C_, Type
Pro	1		173.5 (C)	Pip	1		173.7 (C)
	2	4.88, dd, (8.5, 3.0)	56.7 (CH)		2	4.86, t (6.0)	51.7 (CH)
	3	2.11, m; 1.39, m	28.1 (CH_2_)		3	1.69, m; 1.34, m	23.1 (CH_2_)
	4	1.85, m	24.6 (CH_2_)		4	1.49, m; 1.30, m	17.6 (CH_2_)
	5	3.63, m; 3.29, m	50.1 (CH_2_)		5	1.62, m	22.4 (CH_2_)
					6	3.57, m; 3.40, m	44.9 (CH_2_)
Ant	1		167.4 (C)	Ant	1		171.9 (C)
	2		135.7 (C)		2		136.4 (C)
	3	7.52, d, (7.5)	127.5 (CH)		3	7.35, dd, (8.0, 1.6)	127.9 (CH)
	4	7.16, d, (7.5)	123.2 (CH)		4	7.18, dd, (8.0, 8.0)	123.4 (CH)
	5	7.47, t, (8.0)	130.9 (CH)		5	7.48, dd, (8.0, 8.0)	131.2 (CH)
	6	8.38, d, (8.5)	120.0 (CH)		6	8.40, dd, (8.0, 1.6)	120.2 (CH)
	7		123.9 (C)		7		122.7 (C)
	NH	9.62, s			NH	9.27, s	
Ile	1		168.9 (C)	Ile	1		168.8 (C)
	2	4.38, dd, (8.5, 4.5)	58.1 (CH)		2	4.23, dd, (7.1, 4.2)	58.4 (CH)
	3	2.01, m	36.2 (CH)		3	2.01, m	35.8 (CH)
	4	1.37, m; 1.20, m	24.1 (CH_2_)		4	1.39, m; 1.26, m	24.5 (CH_2_)
	5	0.83, t, (7.0)	11.7 (CH_3_)		5	0.84, t, (9.4)	11.8 (CH_3_)
	3′	0.86, d, (7.0)	15.7 (CH_3_)		3′	0.85, d, (6.8)	15.6 (CH_3_)
	NH	7.20, d, (9.0)			NH	7.26, d, (9.0)	
Gly	1		169.4 (C)	Ala	1		172.2 (C)
	2	4.26, dd, (17.0, 8.0) 3.66, dd, (9.5, 3.5),	42.6 (CH_2_)		2	4.56, m	47.7 (CH)
					3	1.30, d, (7.2)	17.5 (CH_3_)
	NH	7.78, dd, (7.5, 4.0)			NH	7.29, d, (7.5)	
*N*-Me-Phe	1		169.9 (C)	*N*-Me-Phe	1		168.9 (C)
	2	5.30, dd, (12.0, 4.5)	58.8 (CH)		2	5.58, dd, (12.4, 4.4)	57.2 (CH)
	3	3.40, dd, (15.5, 4.5) 3.10, dd, (15.5, 12.5)	32.5 (CH_2_)		3	3.44, m; 3.05, m	31.9 (CH_2_)
	4		137.7 (C)		4		137.8 (C)
	5, 9	7.25, m	128.3 (CH)		5, 9	7.21, d (12.0)	128.2 (CH)
	6, 8	7.31, m	128.3 (CH)		6, 8	7.28, d, (7.2)	128.3 (CH)
	7	7.22, m	126.4 (CH)		7	7.21, t, (7.2)	126.3 (CH_2_)
	*N*-Me	3.00, s	31.6 (CH_3_)		*N*-Me	2.97, s	30.6 (CH_3_)

^a^ Measured in DMSO-*d*_6_ at 500 MHz for ^1^H NMR and 125 MHz for ^13^C NMR. ^b^ Measured in DMSO-*d*_6_ at 400 MHz for ^1^H NMR and 100 MHz for ^13^C NMR.

**Table 2:** ^1^H NMR (500 MHz) and ^13^C NMR (125 MHz) data for compounds **3**–**4**.

	3 ^c^				4 ^d^		
Unit	Position	*δ*_H_, (*J* in Hz)	*δ*_C_, Type	Unit	Position	*δ*_H_, (*J* in Hz)	*δ*_C_, Type
Pro	1		173.5 (C)	Pro	1		176.6 (C)
	2	4.90, dd, (9.0, 3.5)	56.8 (CH)		2	4.88, m	57.9 (CH)
	3	2.11, m; 1.37, m	28.1 (CH_2_)		3	2.03, m; 1.40, m	29.5 (CH_2_)
	4	1.84, m	24.5 (CH_2_)		4	2.08, m; 1.89, m	26.1 (CH_2_)
	5	3.63, m; 3.28, m	50.1 (CH_2_)		5	3.80, m; 3.52, m	52.6 (CH_2_)
Ant	1		167.4 (C)	Ant	1		170.4 (C)
	2		135.6 (C)		2		138.8 (C)
	3	7.52, d, (6.5)	127.4 (CH)		3	7.58, dd (7.5, 1.5)	129.6(CH)
	4	7.18, t, (7.0)	123.2 (CH)		4	7.23, t (7.0)	127.8(CH)
	5	7.47, td, (8.5, 1.0)	130.9 (CH)		5	7.48, td (7.5, 1.5)	132.9(CH)
	6	8.35, d, (8.0)	120.1 (CH)		6	8.50, dd (8.5, 1.0)	121.6(CH)
	7		124.1 (C)		7		123.6(C)
	NH	9.62, s		Abu	1		171.7 (C)
Val	1		168.9 (C)		2	4.42, dd (10.0, 4.0)	57.6 (CH)
	2	5.29, dd, (12.0, 4.5)	58.7 (CH)		3	2.13, m; 1.73, m	25.1 (CH_2_)
	3	2.31, m	29.4 (CH)		4	1.00, t (7.0)	11.1 (CH_3_)
	4	0.88, d, (7.0)	19.0 (CH_3_)	Ala	1		174.8 (C)
	5	0.86, d, (7.0)	16.9 (CH_3_)		2	4.81, dd (14.5, 7.0)	49.6 (CH)
	NH	7.12, d, (9.0)			3	1.44, d (7.0)	18.2 (CH_3_)
Gly	1		169.4 (C)	*N*-Me-Phe	1		172.1 (C)
	2	4.24, dd, (17.0, 8.0), 3.67, dd, (17.0, 4.5),	42.6 (CH_2_)		2 3 4	5.73, dd (12.0, 4.5) 3.63, dd (15.0, 4.0), 3.06, dd (15.0, 12.5)	59.6 (CH) 33.9 (CH_2_) 138.2 (C)
	3	3.63, dd (15.0, 4.0), 3.06, dd (15.0, 12.5)	33.9 (CH_2_)				
	NH	7.79, dd, (8.0, 4.5)			5, 9		129.5 (CH)
*N*-Me-Phe	1		170.0 (C)		7		124.4 (CH)
	2	4.36, dd, (9.0, 4.5)	58.7 (CH)		6, 8		129.5 (CH)
	3	3.41, dd, (15.5, 4.5); 3.12, dd, (15.0, 12.0),	32.5 (CH_2_)		*N*-Me		32.1 (CH_3_)
	4		137.6 (C)				
	5, 9	7.31, m; 7.25, m	128.3(CH)				
	6, 8	7.31, m; 7.25, m	128.4(CH)				
	7	7.23, t, (7.5)	126.4 (CH)				
	*N*-Me	3.01, s	31.5 (CH_3_)				

**^c^** Obtained in DMSO-*d*_6_; ^d^ obtained in CD_3_OD.

## Data Availability

The data presented in this study are available upon request from the corresponding author.

## Supplementary Materials

The following supporting information can be downloaded at https://www.mdpi.com/article/10.3390/md22060282/s1: Figure S1: HPLC and UV spectra of *Aspergillus fumigatus* GXIMD 03099; Figures S2–S12: ^1^H, ^13^C, DEPT, HSQC, HMBC, ^1^H–^1^H COSY, NOESY, HR-ESI-MS, Marfey’s analysis, and CD spectrum for compound **1;** Figures S13–S23: ^1^H, ^13^C, DEPT, HSQC, HMBC, ^1^H–^1^H COSY, NOESY, HR-ESI-MS, Marfey’s analysis, and CD spectrum for compound **2;** Figures S24–S34: ^1^H, ^13^C, DEPT, HSQC, HMBC, ^1^H–^1^H COSY, NOESY, HR-ESI-MS, Marfey’s analysis, and CD spectrum for compound **3**; Figures S35–S45: ^1^H, ^13^C, DEPT, HSQC, HMBC, ^1^H–^1^H COSY, NOESY, HR-ESI-MS, Marfey’s analysis, and CD spectrum for compound **4**; crystal data and structure refinement for compound **5**.
