# Supplementary material for: New Cyclic Pentapeptides from the Mangrove-Derived Aspergillus fumigatus GXIMD 03099
Source: Mar Drugs. 2024 Jun 16;22(6):282. doi: 10.3390/md22060282 (PMC11204760; doi:10.3390/md22060282)
Supplement: Supplementary file 1 [file marinedrugs-22-00282-s001.zip › marinedrugs-3053021-supplementary.pdf]

Supplementary Material
New Cyclic pentapeptides from the Mangrove-derived *Aspergillus fumigatus* GXIMD 03099

Yu Wang[1,2,†], Guangping Cao[1,2,†], Yuman Gan[1,2], Xiao Lin[1,2], Xiangxi Yi[1,2], Longyan Zhao[1,2], Yonghong Liu[1,2], Chenghai Gao[1,2,*], and Meng Bai[1,2,*]

1 Guangxi Key Laboratory of Marine Drugs, Guangxi University of Chinese Medicine, Nanning 530200, China
2 Institute of Marine Drugs, Guangxi University of Chinese Medicine, Nanning 530200, China
* Correspondence: gaoch@gxtcmu.edu.cn (C.G.); XXBai2014@163.com.
† These authors contributed equally to this work.

Abstract: Four new cyclic pentapeptides, avellanins D–G (**1**–**4**), together with four known compounds (**5**–**8**), were isolated from a mangrove-derived *Aspergillus fumigatus* GXIMD 03099 fungus from *Acanthus ilicifolius* L. Their structures were elucidated by analysis of HRESIMS, NMR, and ESI-MS/MS data. Their absolute configurations were determined by X-ray diffraction analysis and Marfey's method. Compounds **1**–**8** were screened for insecticidal and antibacterial activities. Compound **2** showed insecticidal activity against newly hatched larvae of *Culex quinquefasciatus* with an LC_{50} value of 86.6 μM; compound **4** had weak activity against *Vibrio harveyi* with an MIC value of 5.85 μM.

List of Supporting Information

Figure S1. HPLC and UV spectra of *Aspergillus fumigatus* GXIMD 03099.

Figure S2. ^{1}H NMR (DMSO-d_6, 500 MHz) spectrum of **1**.

Figure S3. ^{13}C NMR (DMSO-d_6, 125 MHz) spectrum of **1**.

Figure S4. DEPT (DMSO-d_6, 125 MHz) spectrum of **1**.

Figure S5. HMQC spectrum of **1**.

Figure S6. HMBC spectrum of **1**.

Figure S7. ^{1}H-^{1}H COSY spectrum of **1**.

Figure S8. NOESY spectrum of **1**.

Figure S9. HR-ESI-MS spectrum of **1**.

Figure S10. ESI-MS/MS spectrum of **1**.

Figure S11. Marfey's analysis of **1**.

Figure S12. CD spectrum of **1** in MeOH.

Figure S13. ^{1}H NMR (DMSO-d_6, 400 MHz) spectrum of **2**.

Figure S14. ^{13}C NMR (DMSO-d_6, 100MHz) spectrum of **2**.

Figure S15. DEPT (DMSO-d_6, 100 MHz) spectrum of **2**.

Figure S16. HMQC spectrum of **2**.

Figure S17. HMBC spectrum of **2**.

Figure S18. ^{1}H-^{1}H COSY spectrum of **2**.

Figure S19. NOESY spectrum of **2**.

Figure S20. HR-ESI-MS spectrum of **2**.

Figure S21. ESI-MS/MS spectrum of **2**.

Figure S22. Marfey's analysis of **2**.

Figure S23. CD spectrum of **2** in MeOH.

Figure S24. ^{1}H NMR (DMSO-d_6, 500 MHz) spectrum of **3**.

Figure S25. ^{13}C NMR (DMSO-d_6, 125 MHz) spectrum of **3**.

Figure S26. DEPT (DMSO-d_6, 125 MHz) spectrum of **3**.

Figure S27. HMQC spectrum of **3**.

Figure S28. HMBC spectrum of **3**.

Figure S29. ^{1}H-^{1}H COSY spectrum of **3**.

Figure S30. NOESY spectrum of **3**.

Figure S31. HR-ESI-MS spectrum of **3**.

Figure S32. ESI-MS/MS spectrum of **3**.

Figure S33. Marfey's analysis of **3**.

Figure S34. CD spectrum of **3** in MeOH.

Figure S35. ^{1}H NMR (CD_3OD, 500 MHz) spectrum of **4**.

Figure S36. ^{13}C NMR (CD_3OD, 125 MHz) spectrum of **4**.

Figure S37. DEPT (CD_3OD, 125 MHz) spectrum of **4**.

Figure S38. HMQC spectrum of **4**.

Figure S39. HMBC spectrum of **4**.

Figure S40. ^{1}H-^{1}H COSY spectrum of **4**.

Figure S41. NOESY spectrum of **4**.

Figure S42. HR-ESI-MS spectrum of **4**.

Figure S43. ESI-MS/MS spectrum of **4**.

Figure S44. Marfey's analysis of **4**.

Figure S45. CD spectrum of **4** in MeOH.

Crystal data and structure refinement for **5**

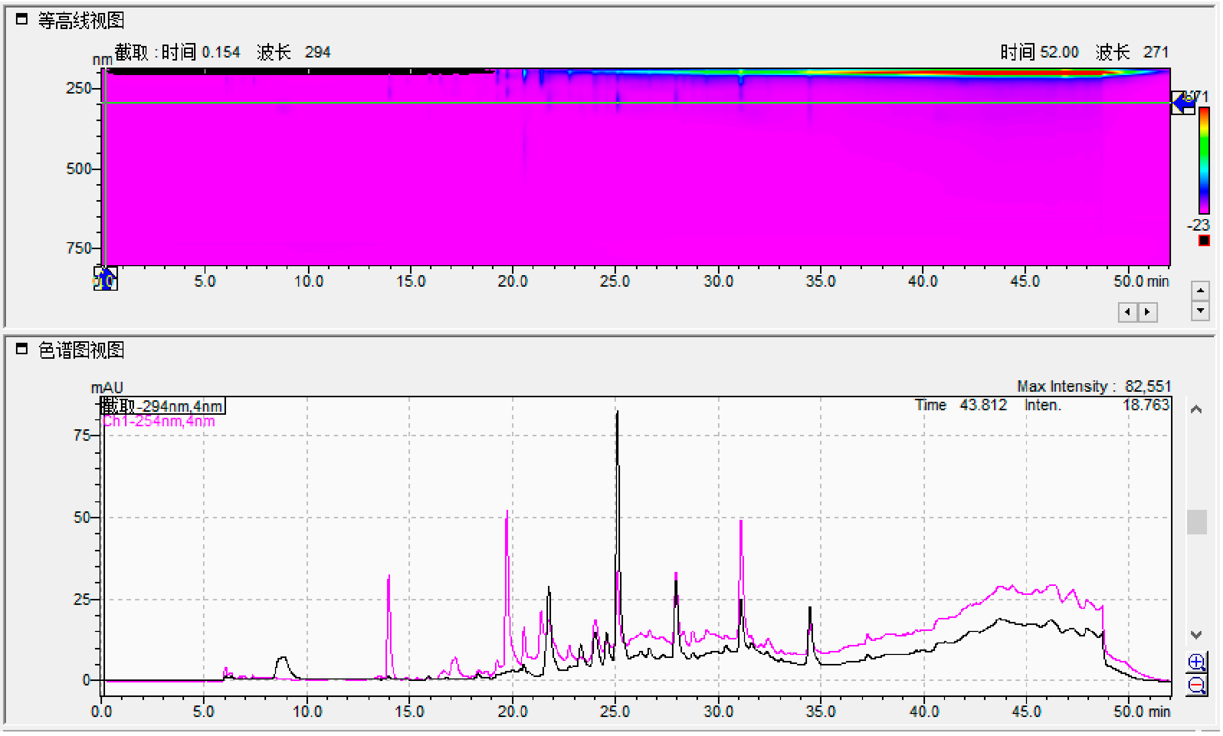

Figure S1. HPLC and UV spectra of *Aspergillus fumigatus* GXIMD 03099.

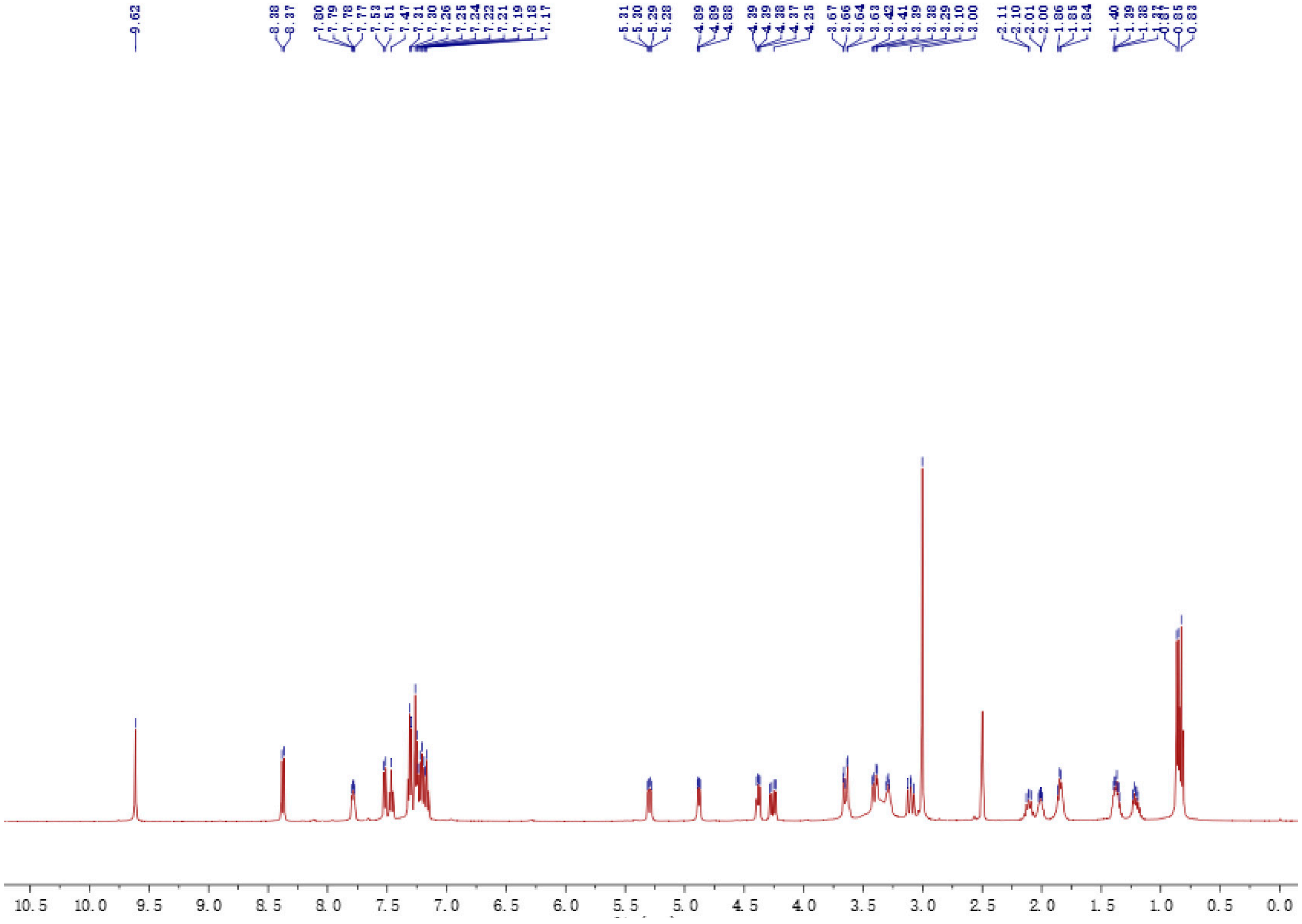

Figure S2. ^{1}H NMR (DMSO-d_6, 500 MHz) spectrum of **1**.

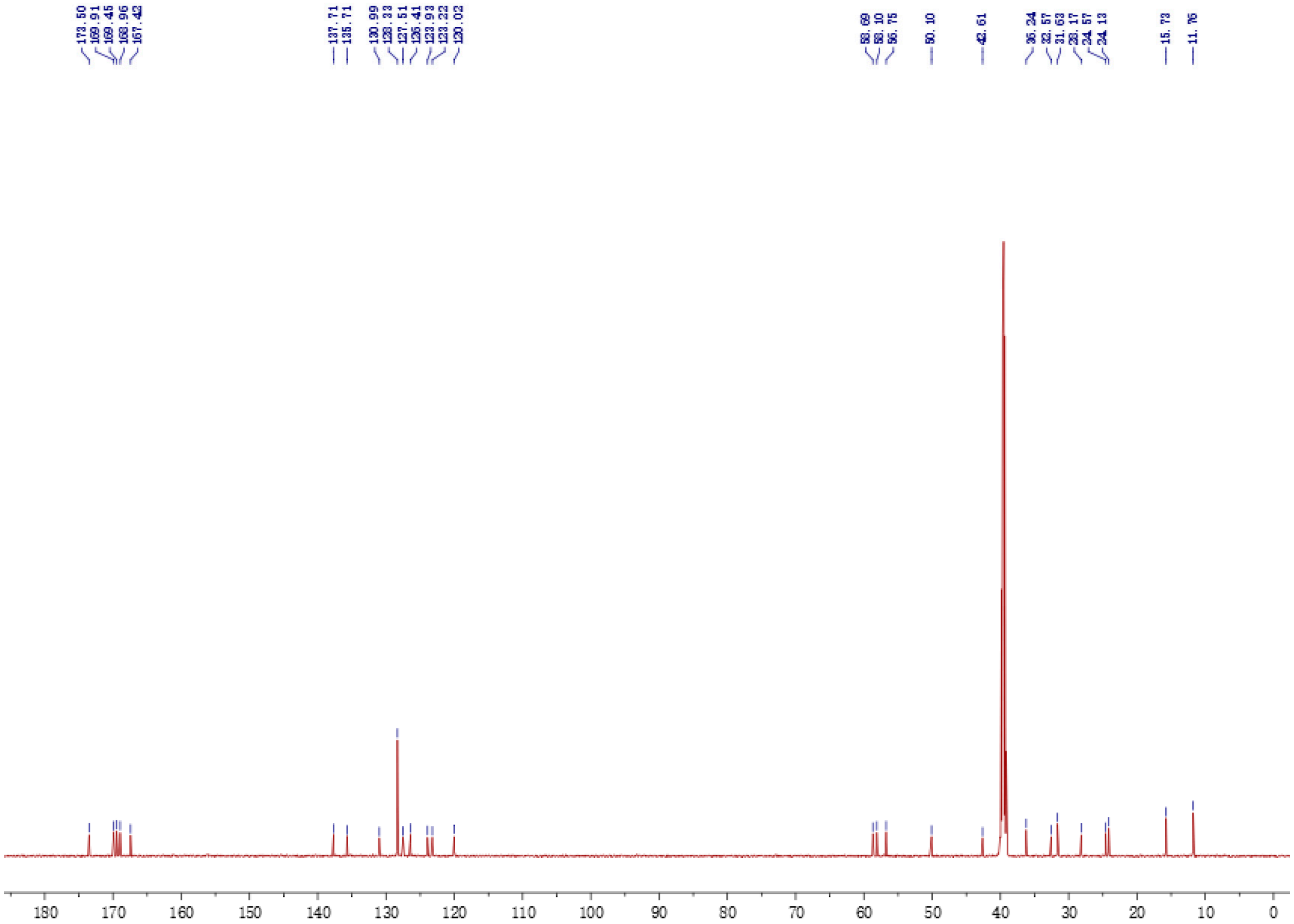

Figure S3. ^{13}C NMR (DMSO-d_6, 125 MHz) spectrum of **1**.

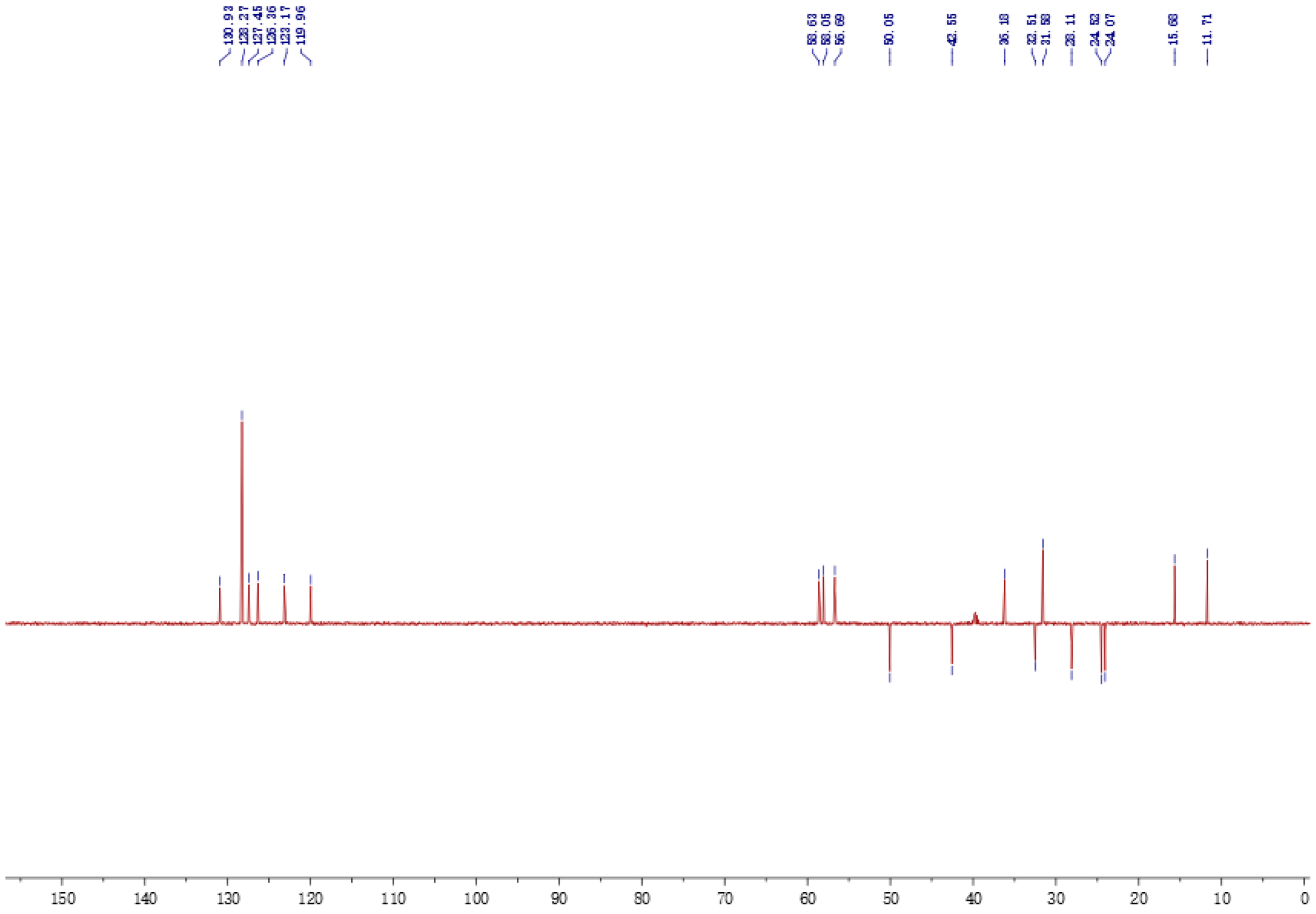

Figure S4. DEPT (DMSO-d_6, 125 MHz) spectrum of **1**.

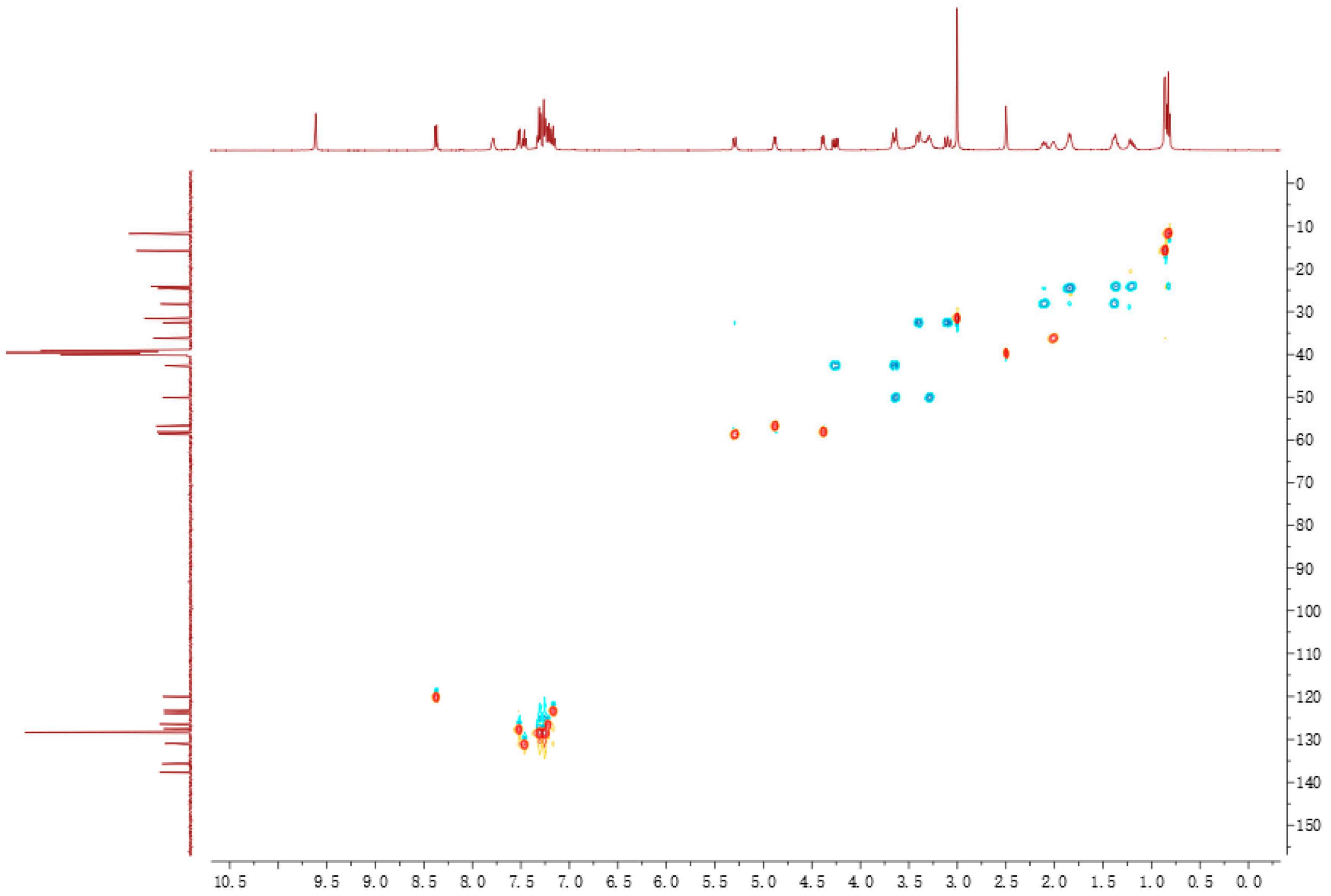

Figure S5. HMQC spectrum of **1**.

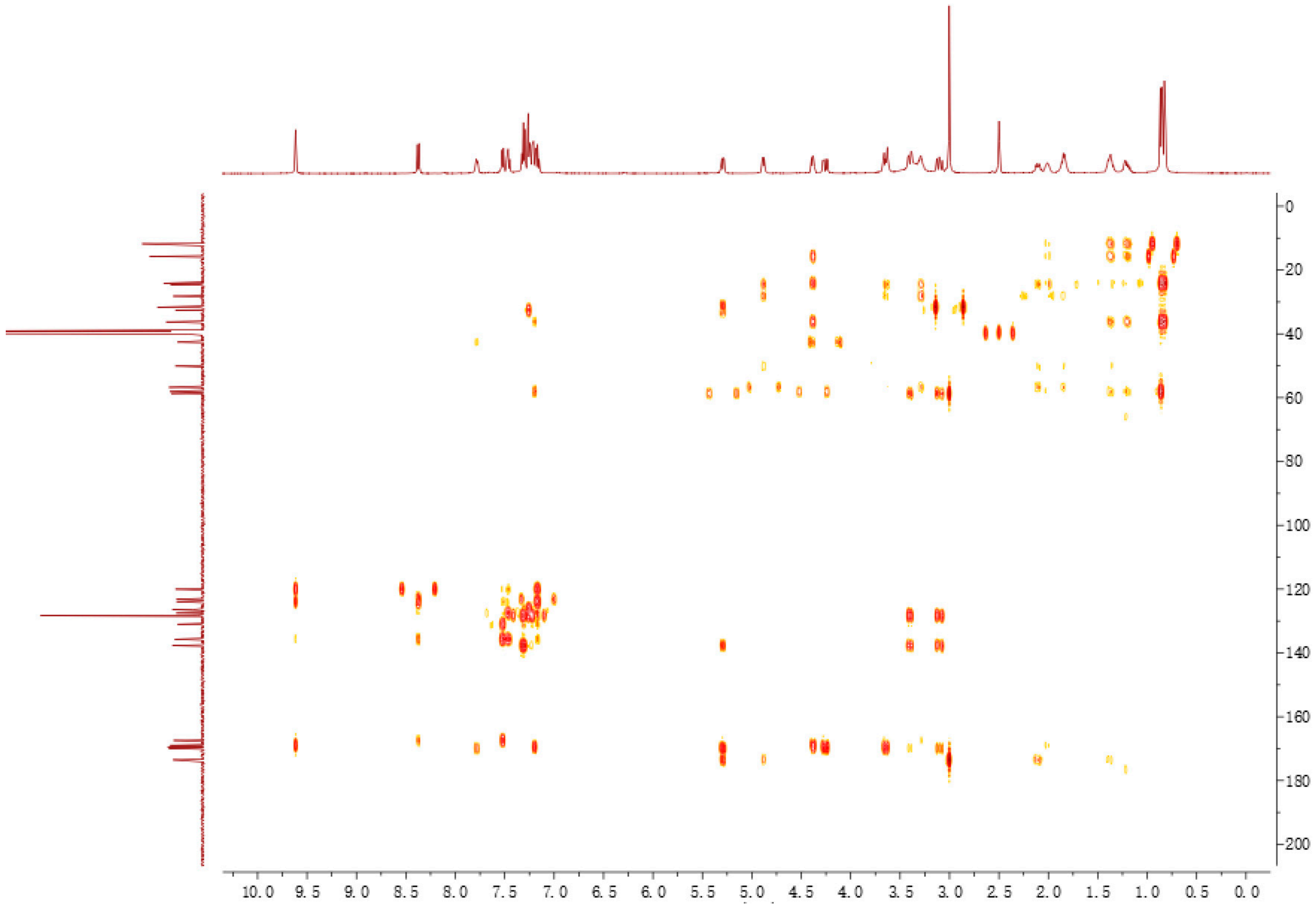

Figure S6. HMBC spectrum of **1**.

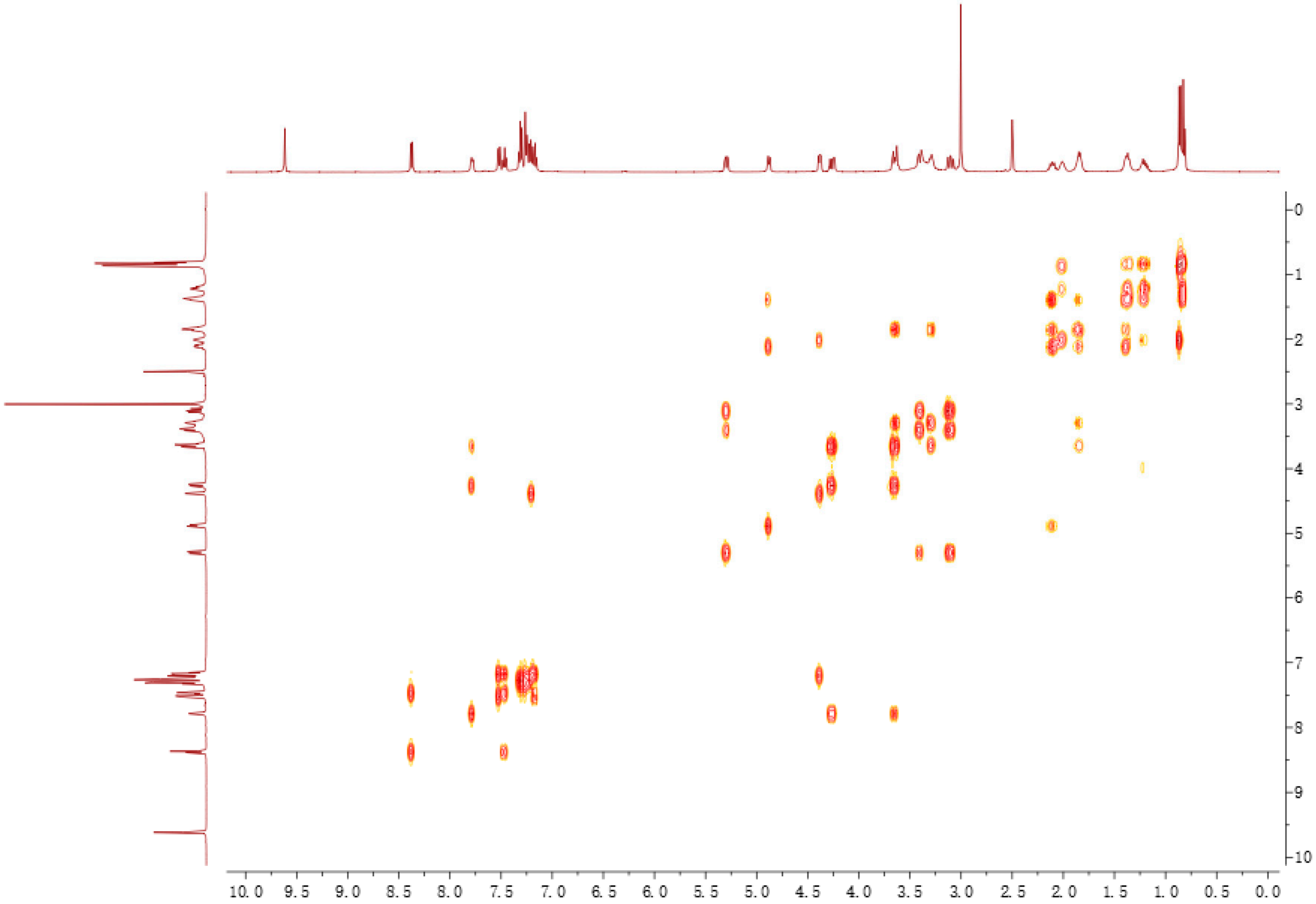

Figure S7. ^{1}H-^{1}H COSY spectrum of **1**.

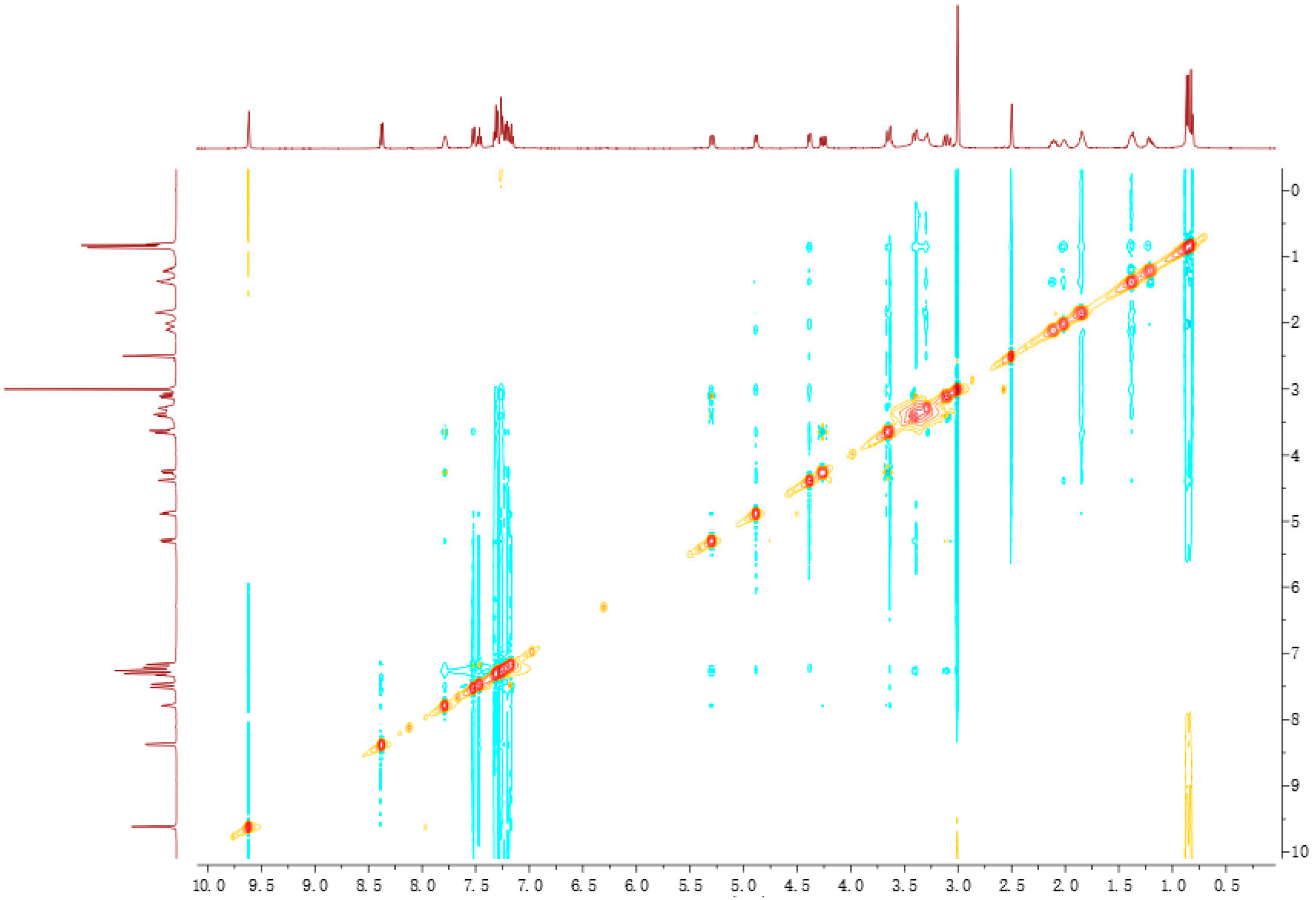

Figure S8. NOESY spectrum of **1**.

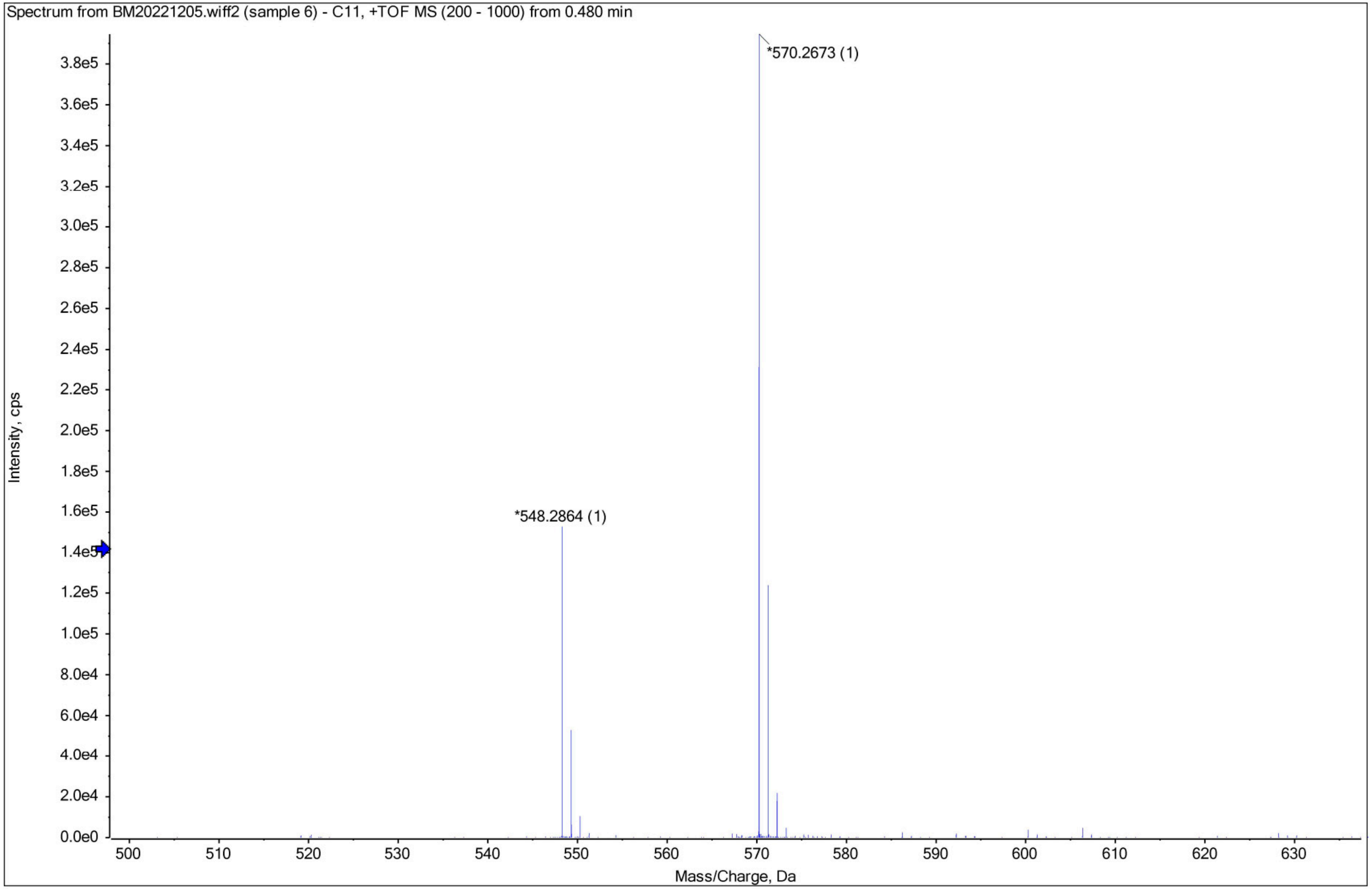

Figure S9. HR-ESI-MS spectrum of **1**.

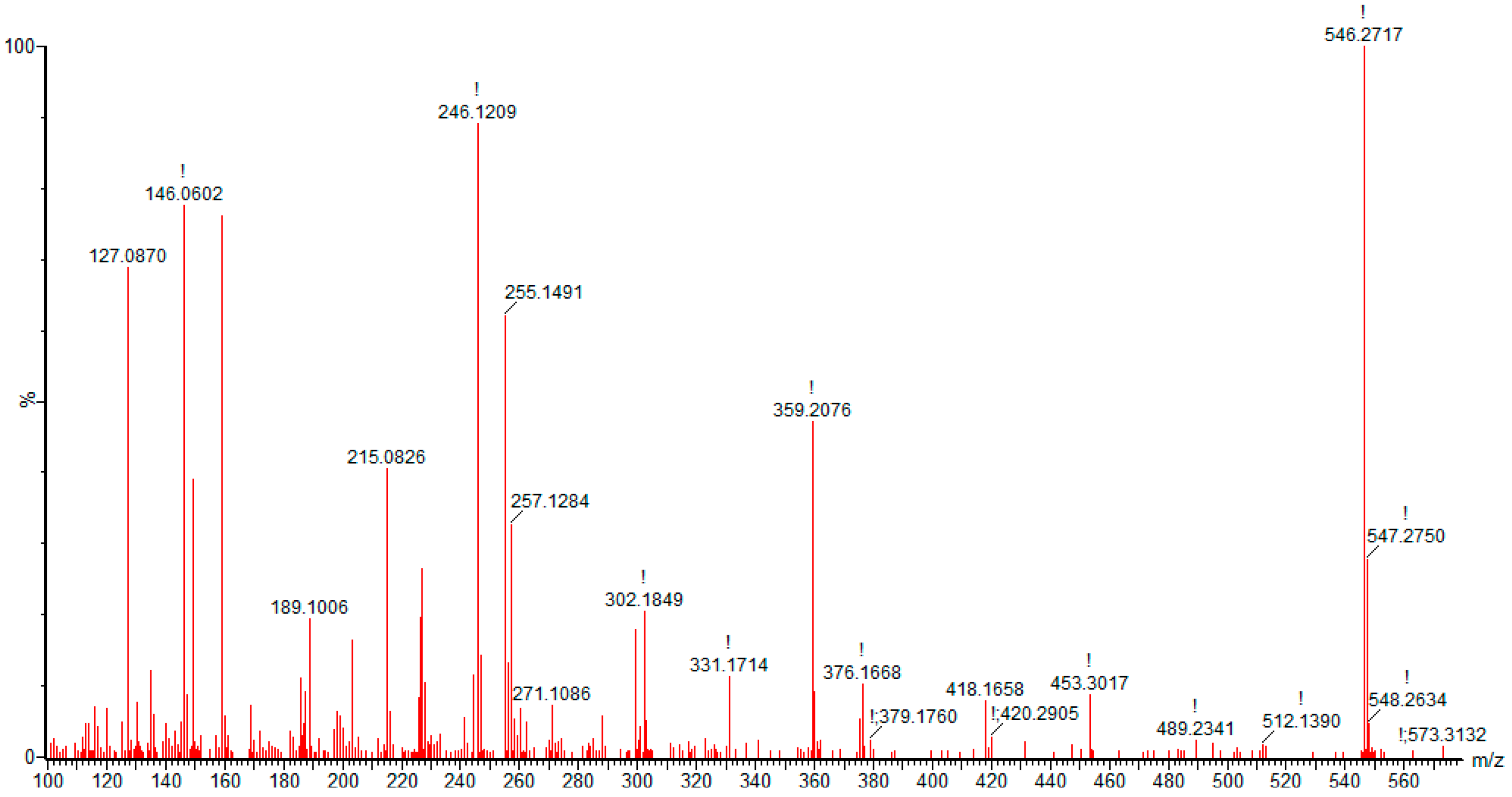

Figure S10. ESI-MS/MS spectrum of **1**

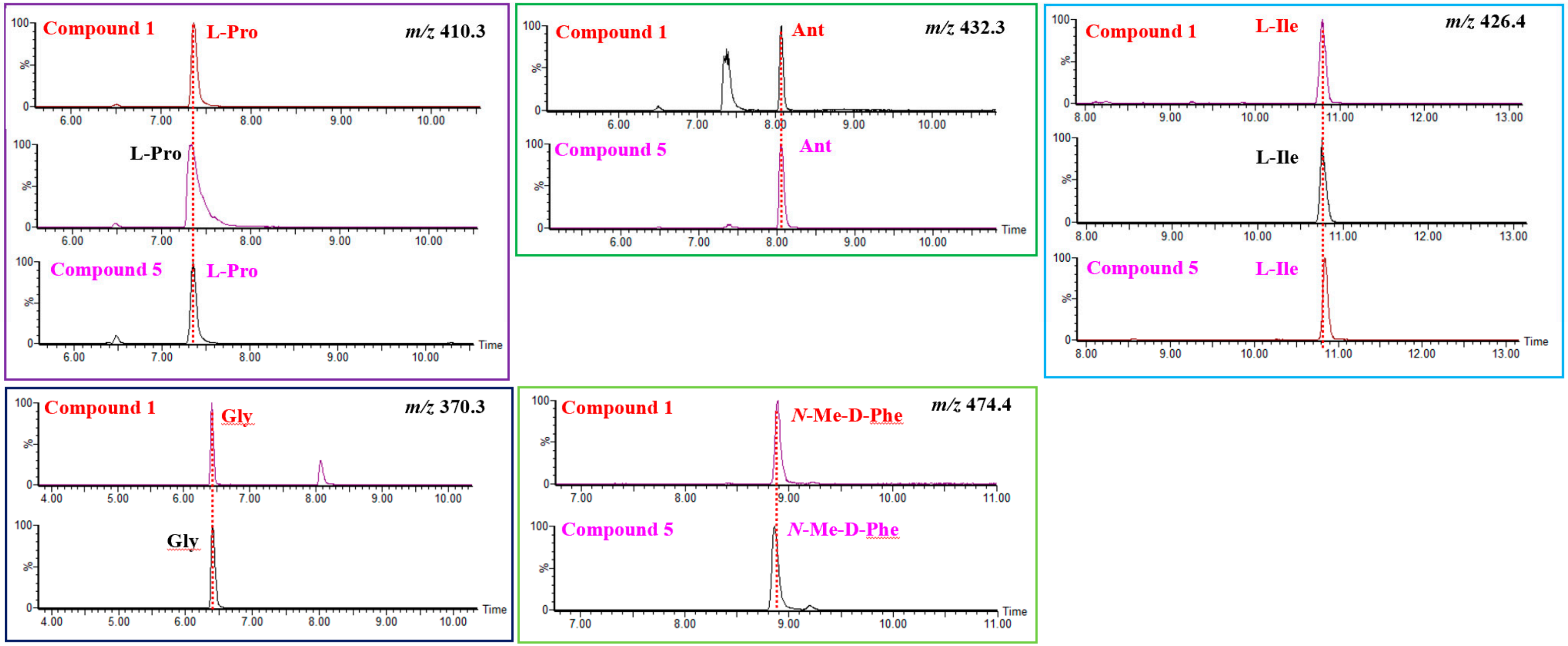

Figure S11. Marfey's analysis of **1.**

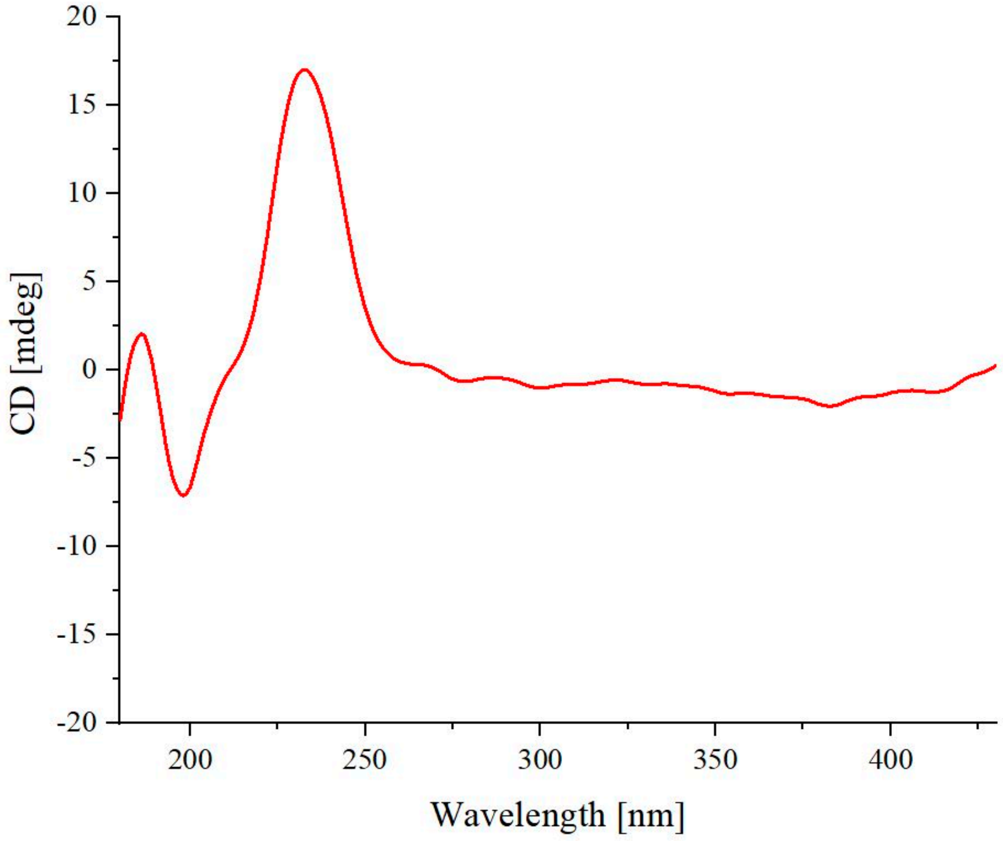

Figure S12. CD spectrum of **1** in MeOH.

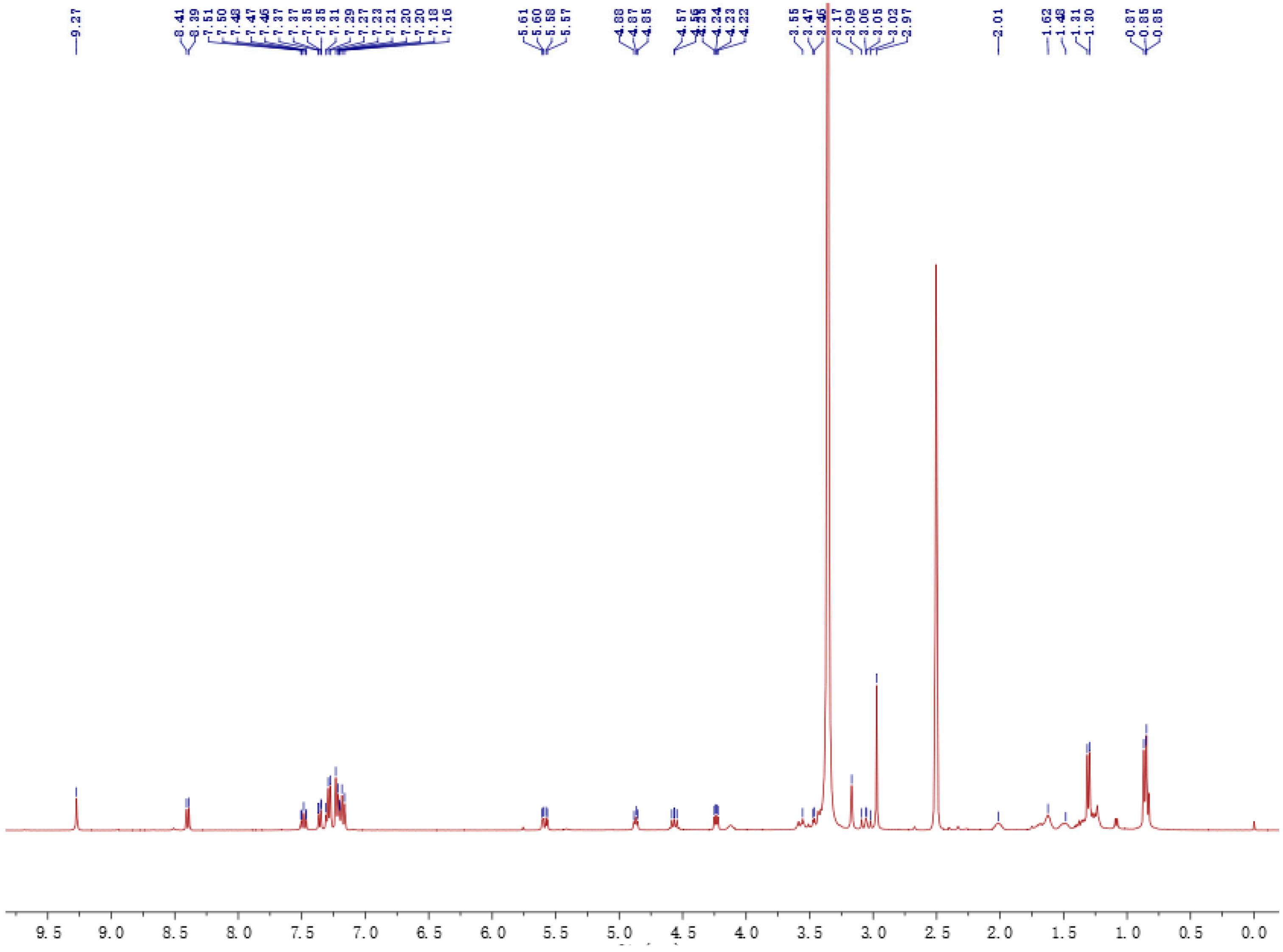

Figure S13. ^{1}H NMR (DMSO-d_6, 400 MHz) spectrum of **2**.

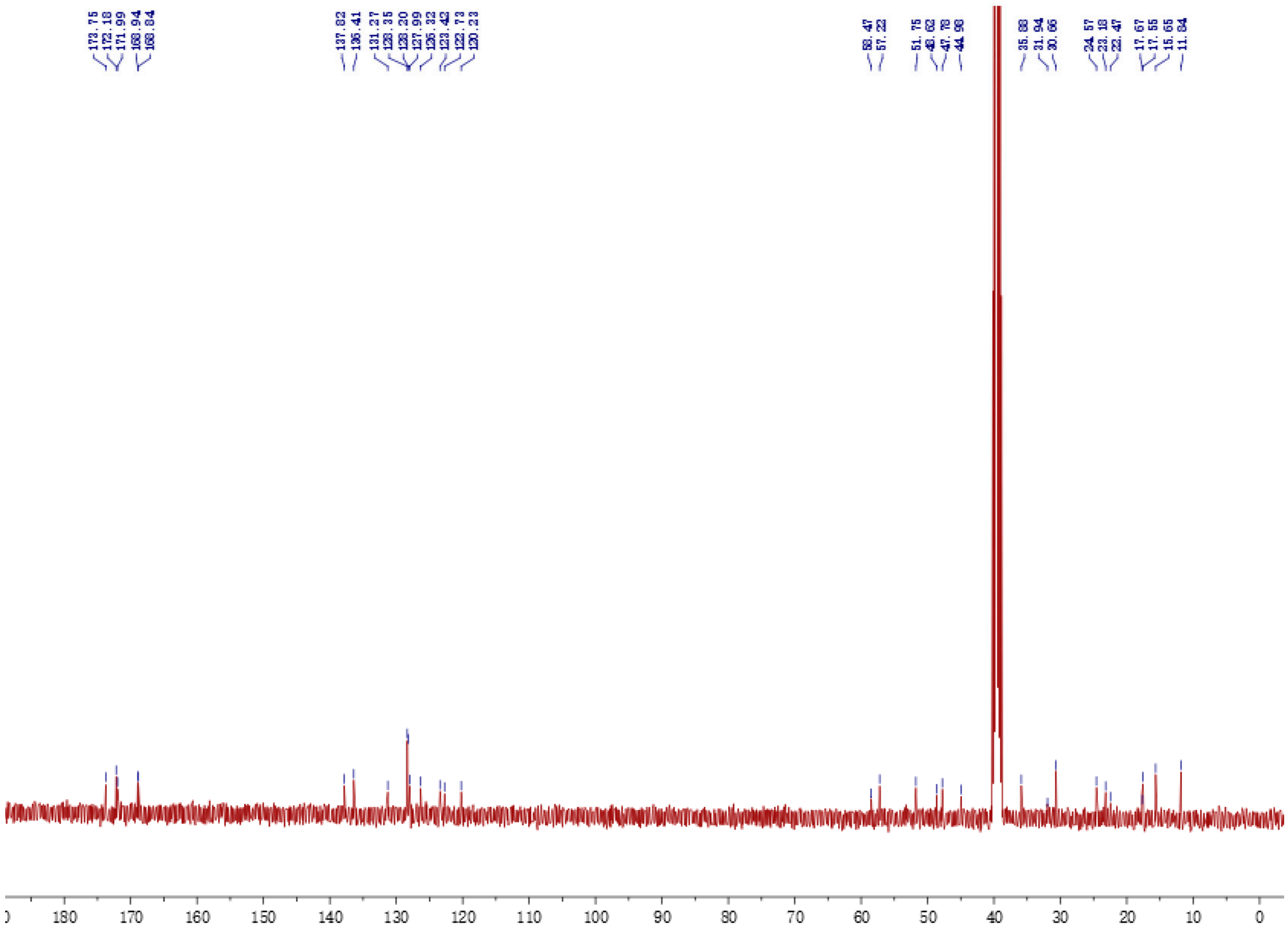

Figure S14. ^{13}C NMR (DMSO-d_6, 100MHz) spectrum of **2**.

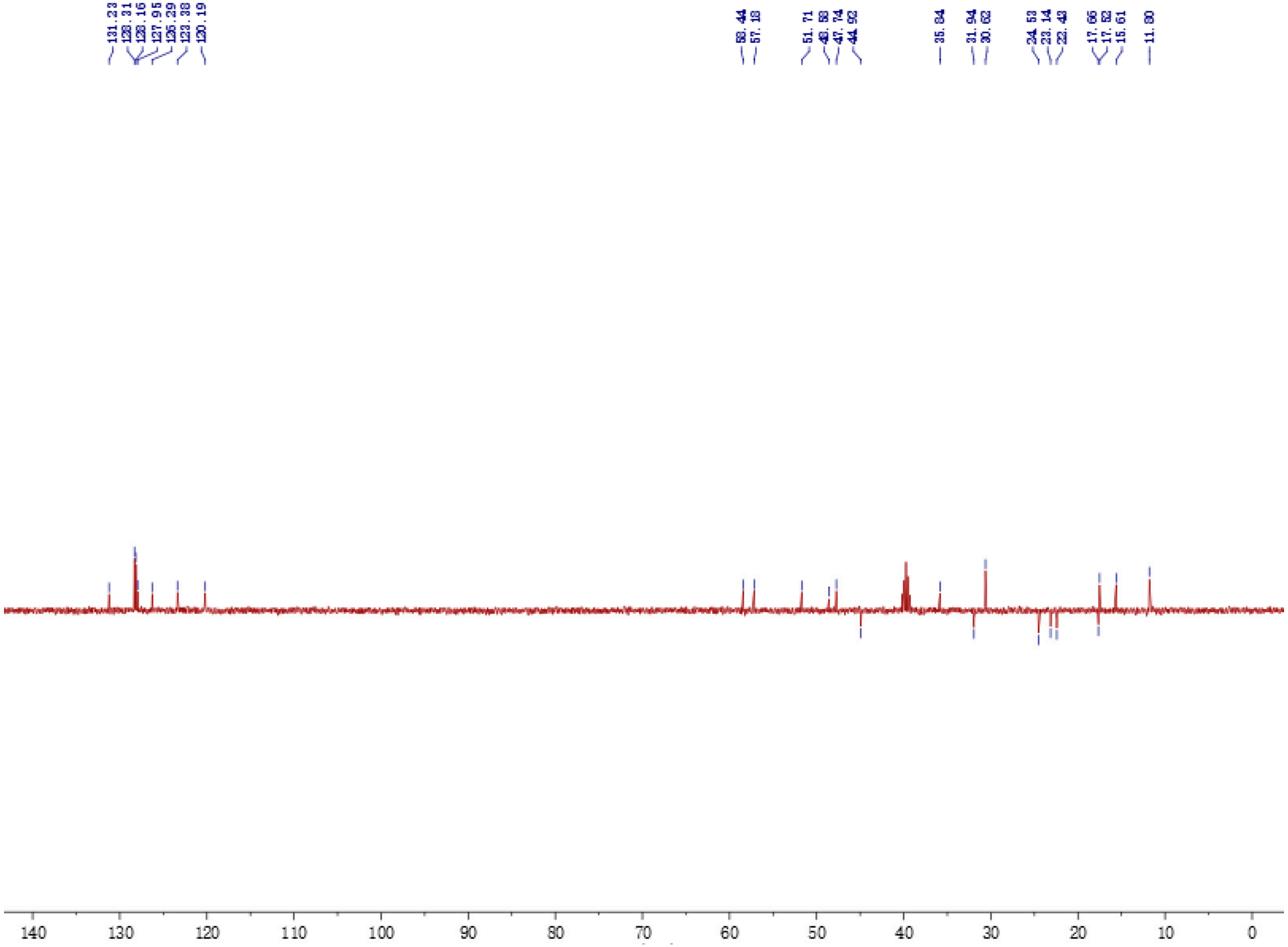

Figure S15. DEPT (DMSO-d_6, 100 MHz) spectrum of **2**.

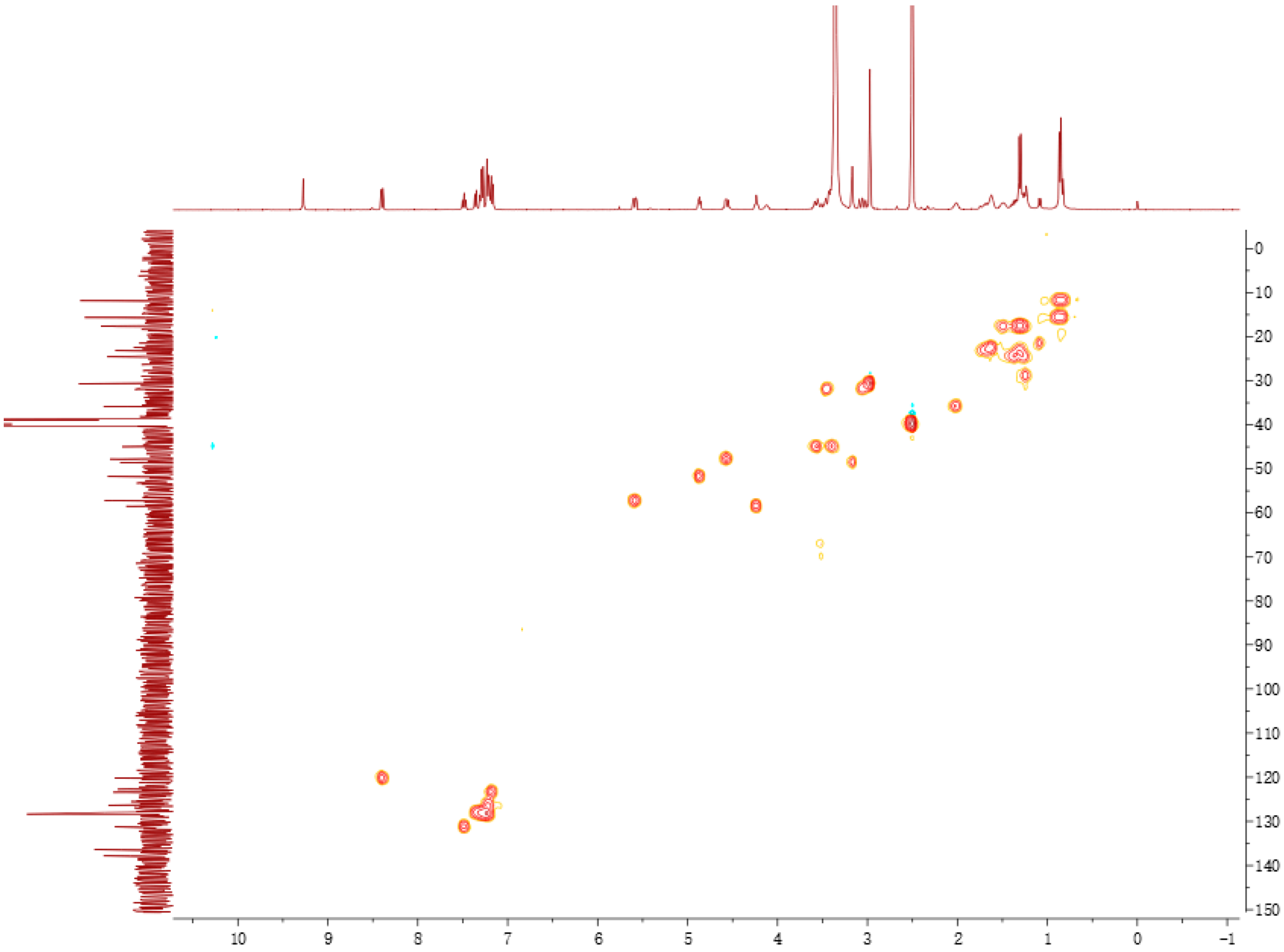

Figure S16. HMQC spectrum of **2**.

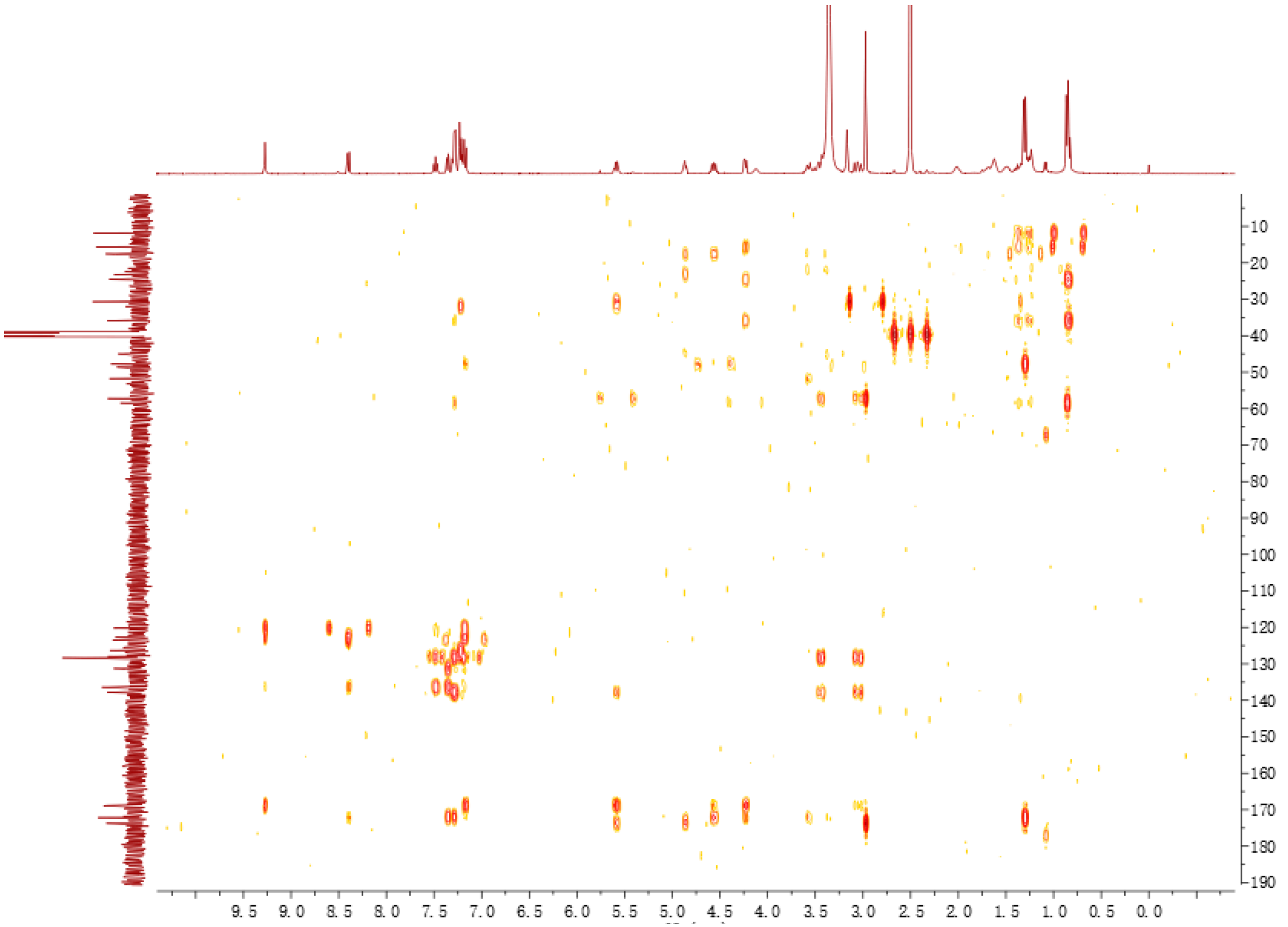

Figure S17. HMBC spectrum of **2**.

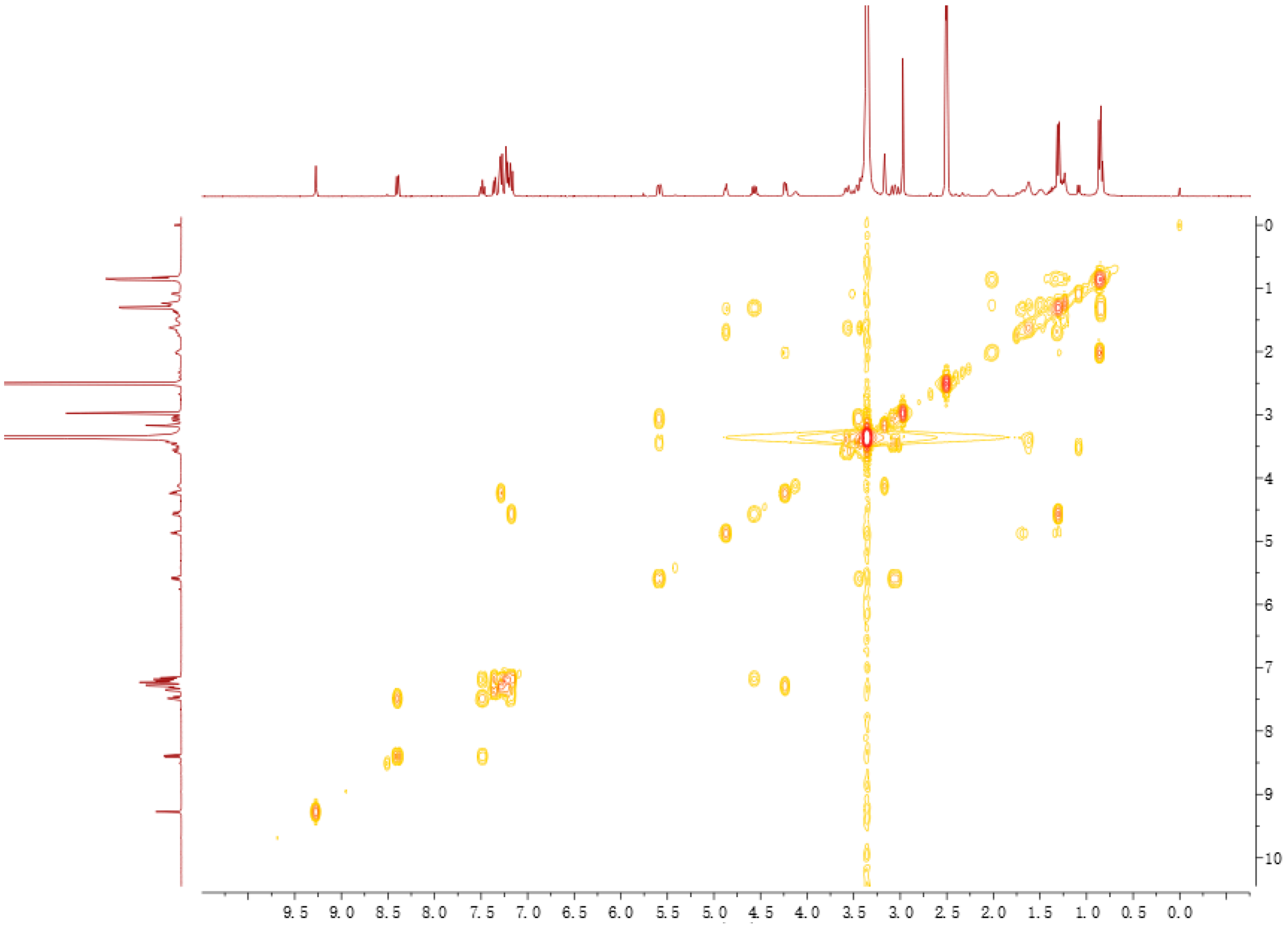

Figure S18. ^{1}H-^{1}H COSY spectrum of **2**.

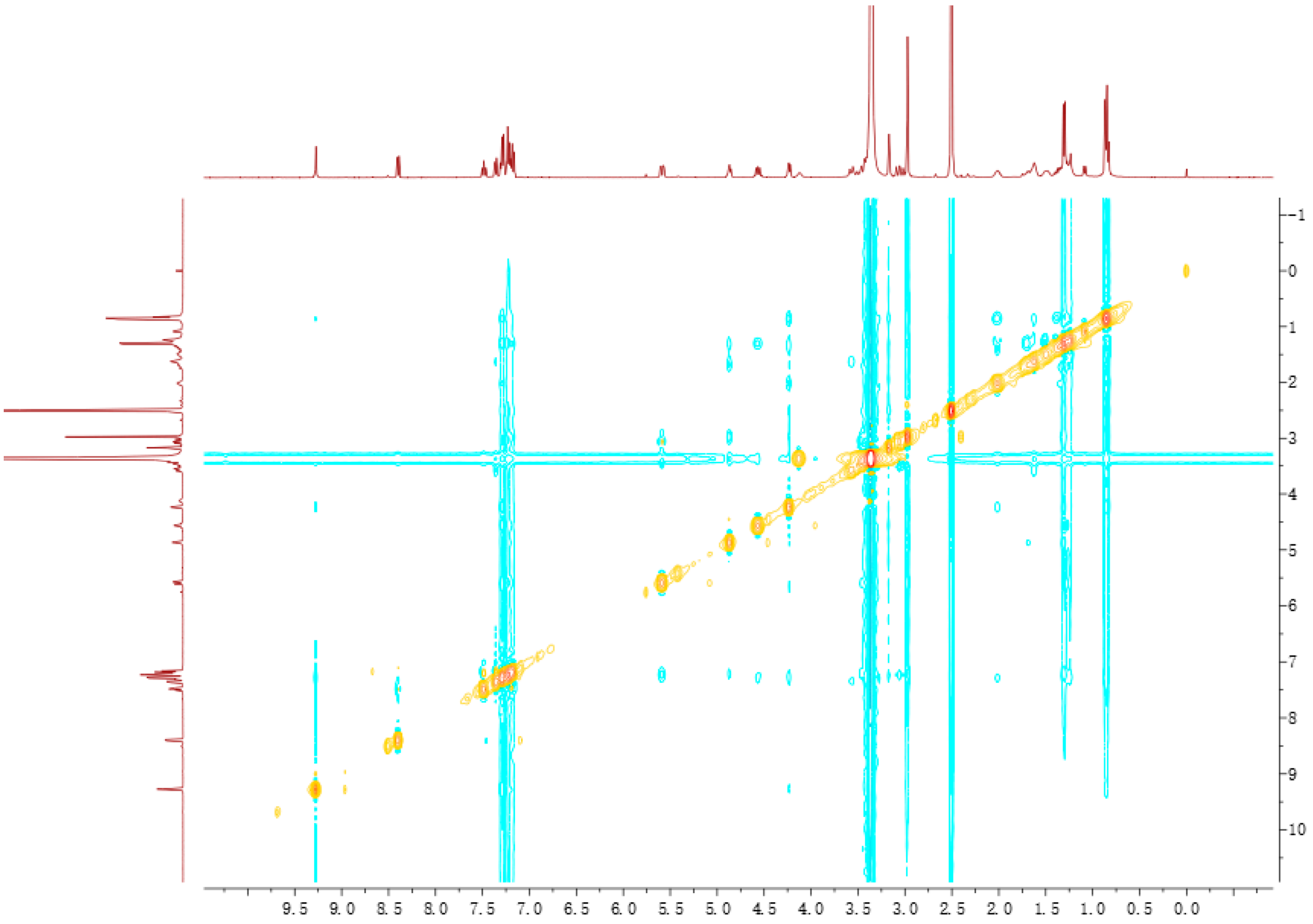

Figure S19. NOESY spectrum of **2**.

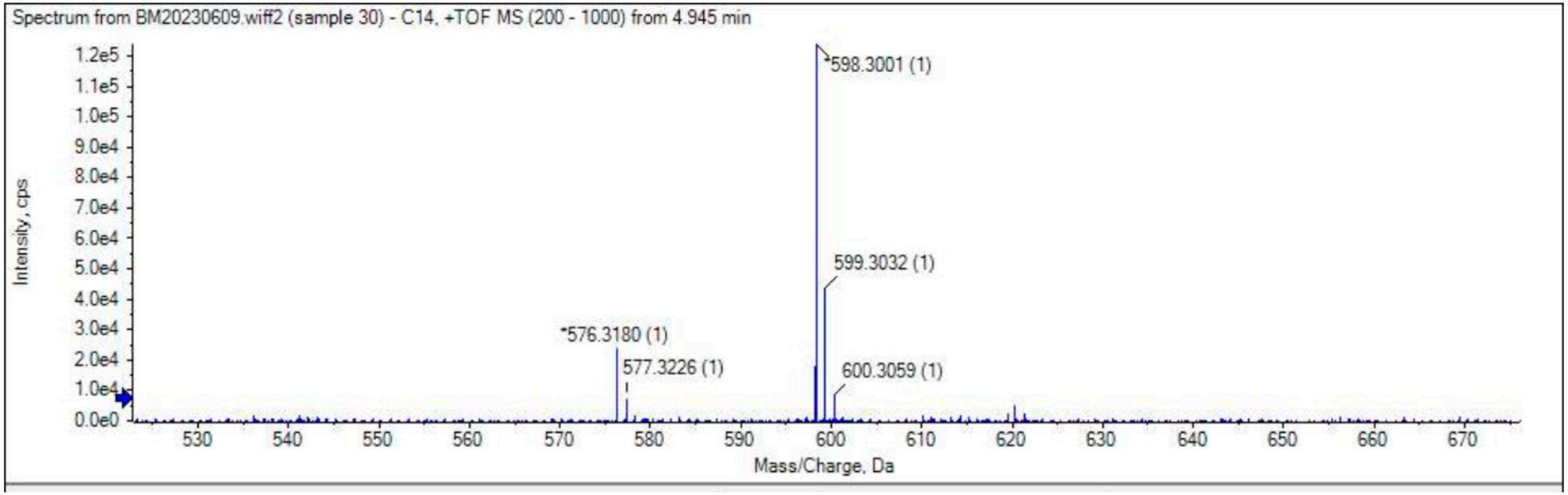

Figure S20. HR-ESI-MS spectrum of **2**.

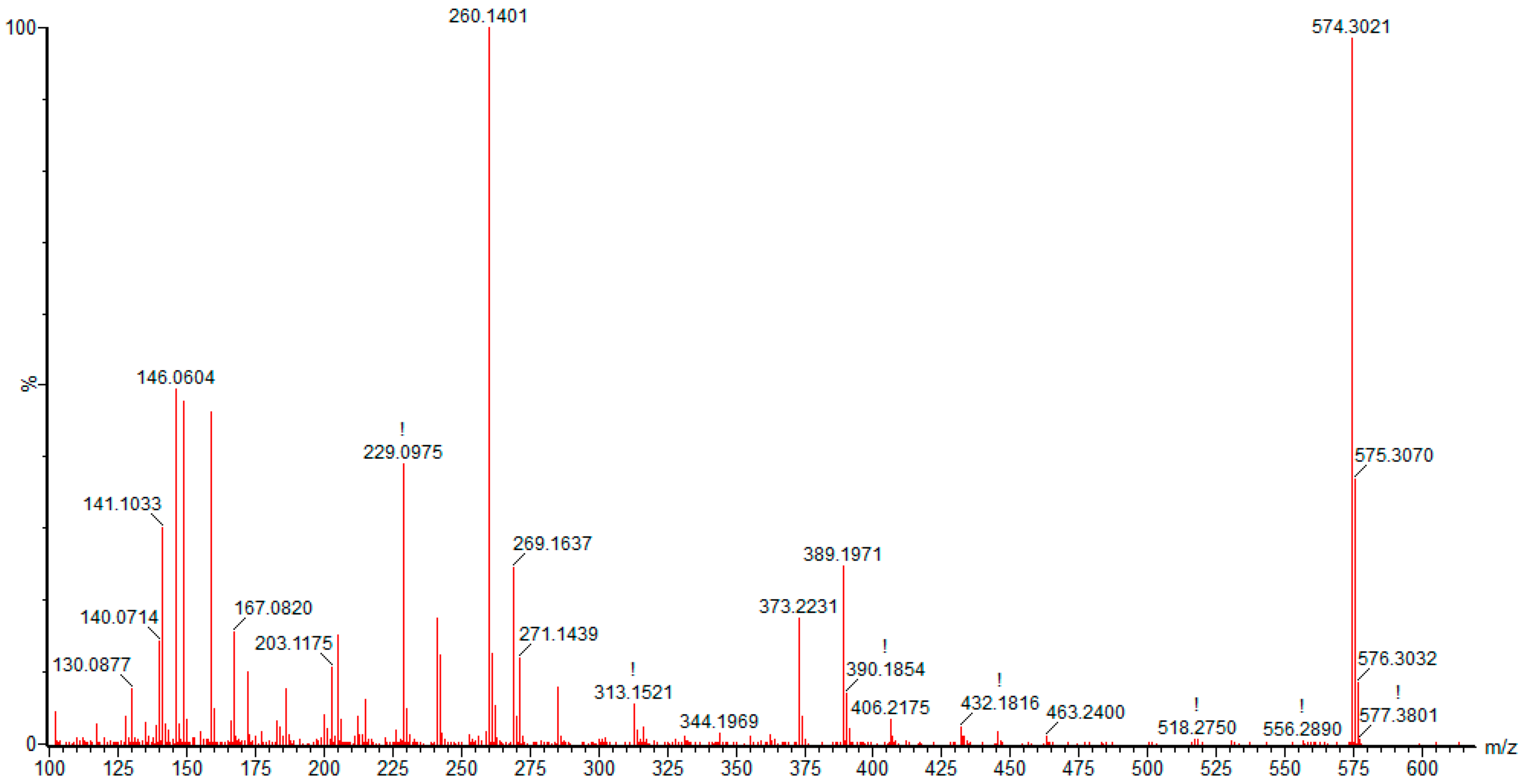

Figure S21. ESI-MS/MS spectrum of **2**.

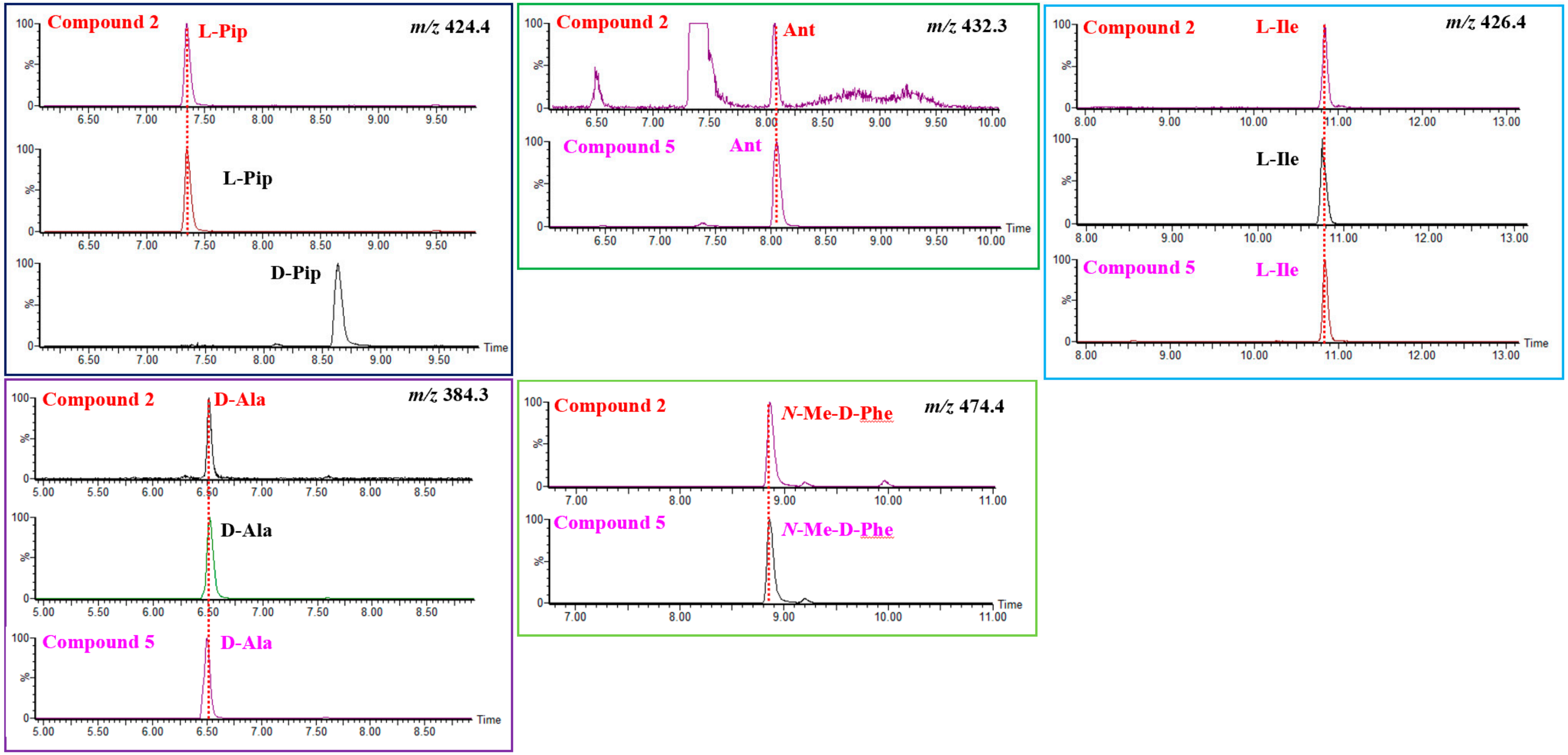

Figure S22. Marfey's analysis of **2**.

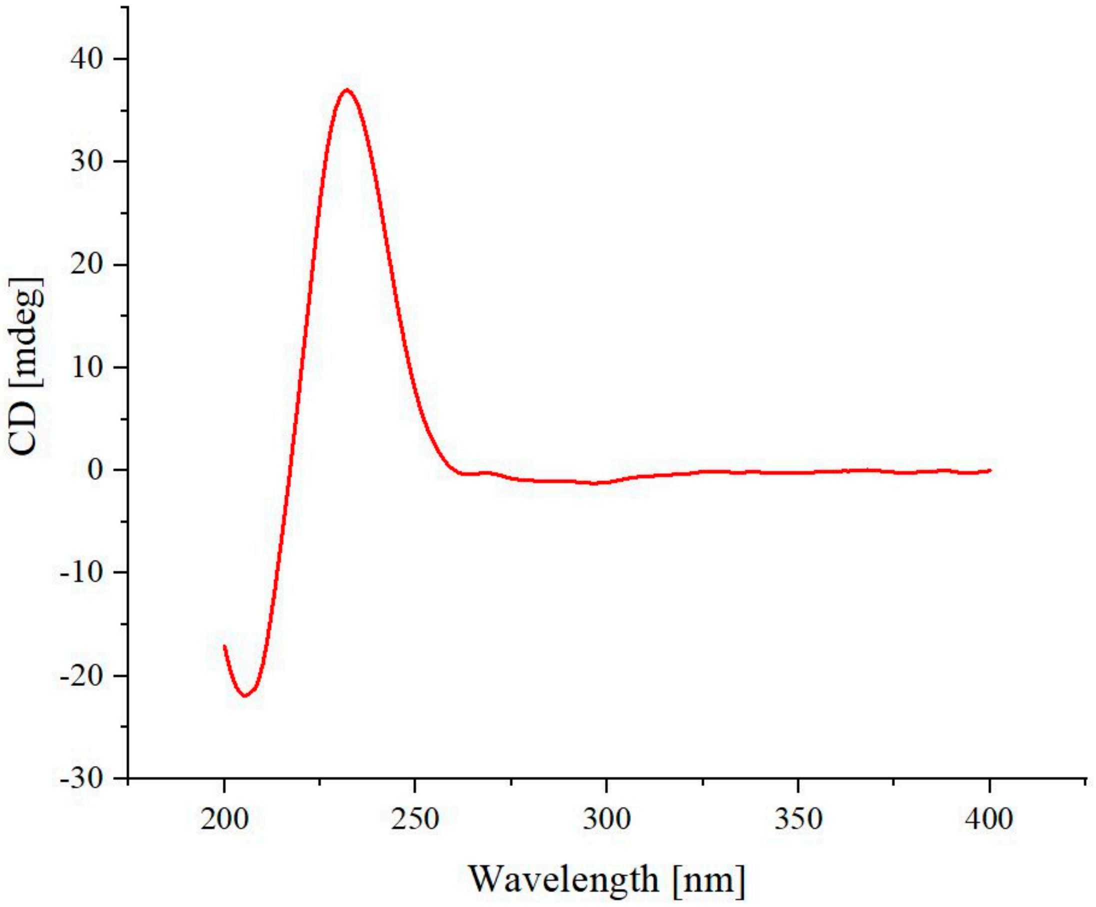

Figure S23. CD spectrum of **2** in MeOH.

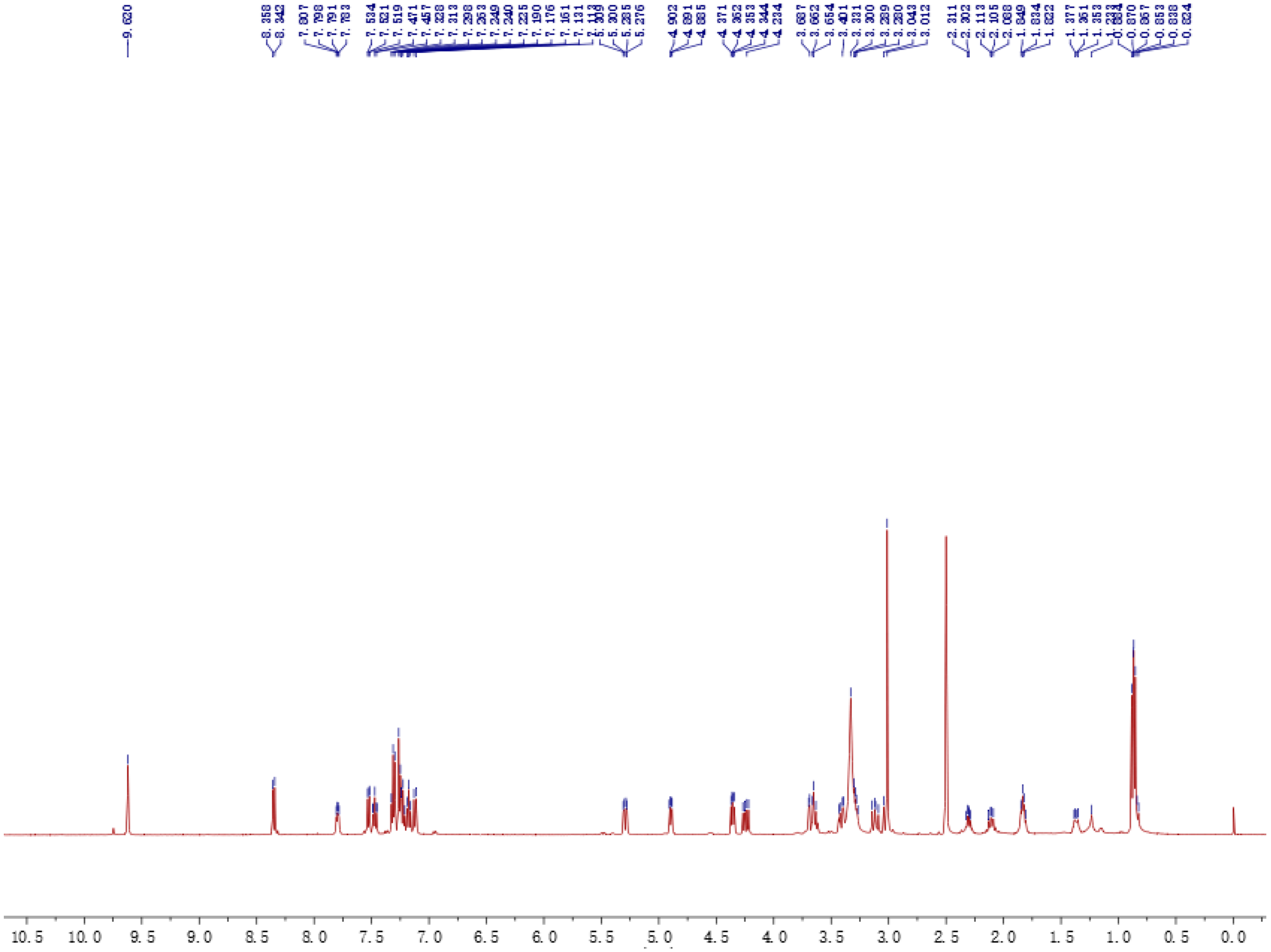

Figure S24. ^{1}H NMR (DMSO-d_6, 500 MHz) spectrum of **3**.

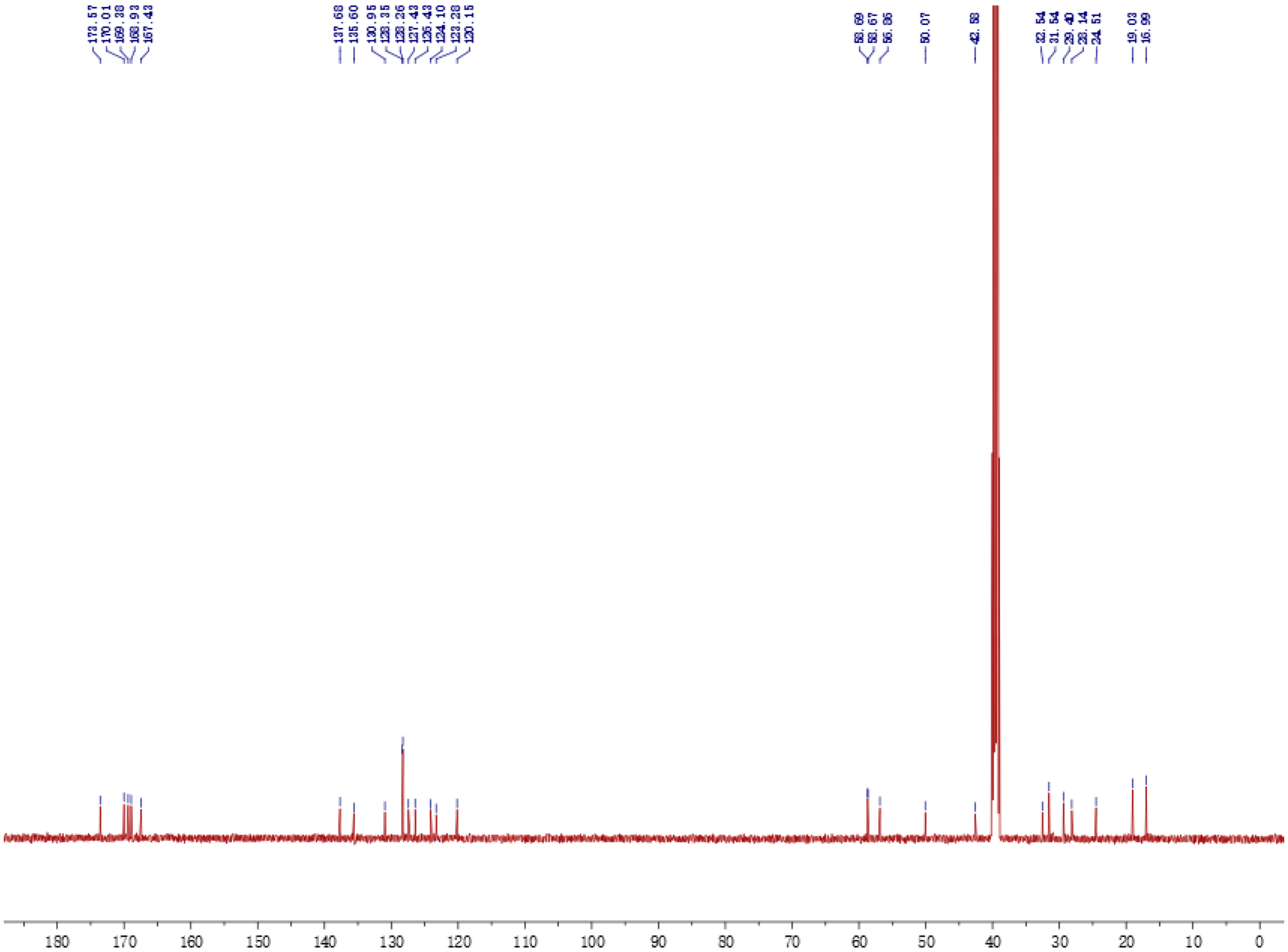

Figure S25. ¹³C NMR (DMSO-d_6, 125 MHz) spectrum of **3**.

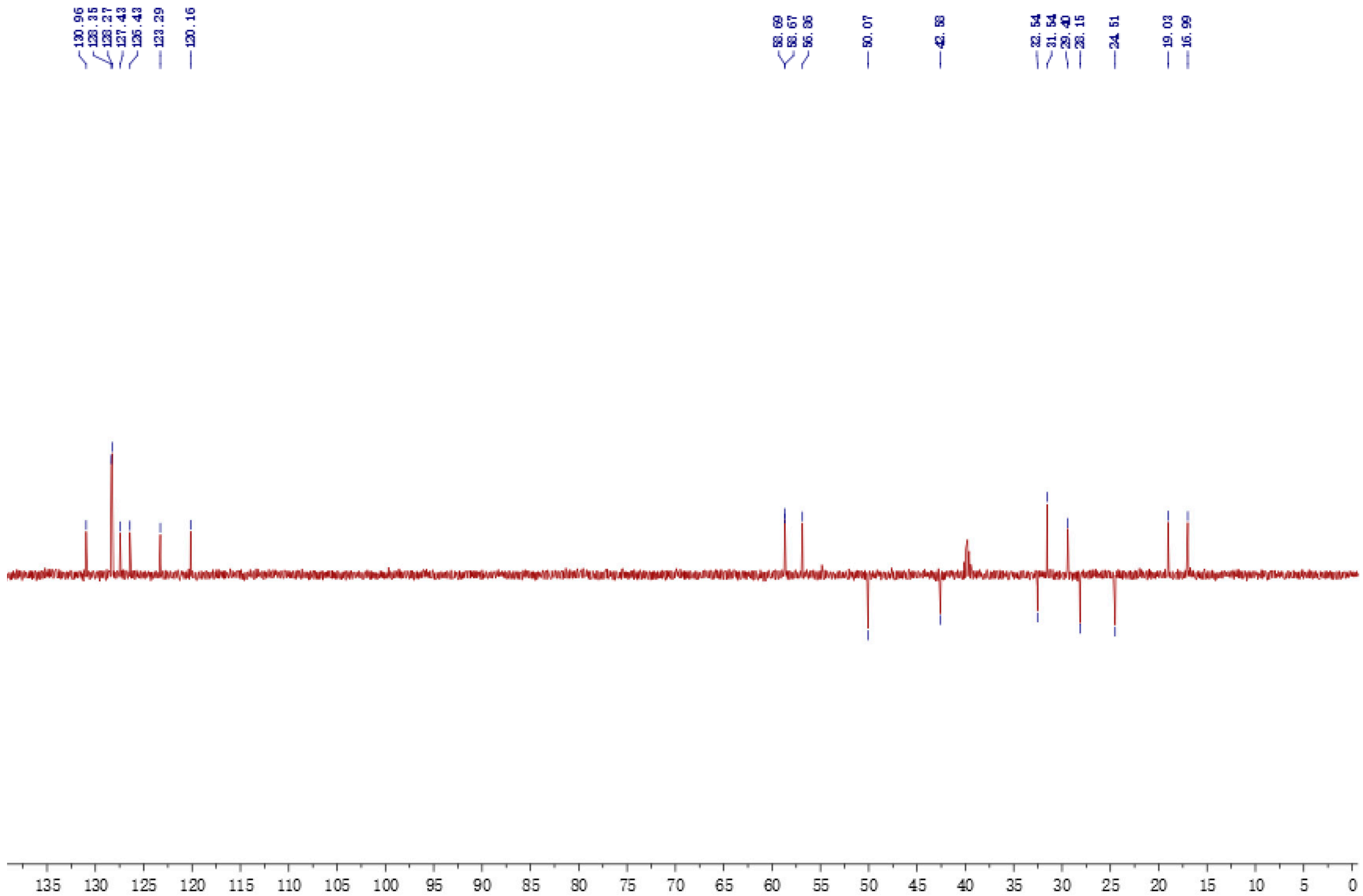

Figure S26. DEPT (DMSO-d_6, 125 MHz) spectrum of **3**.

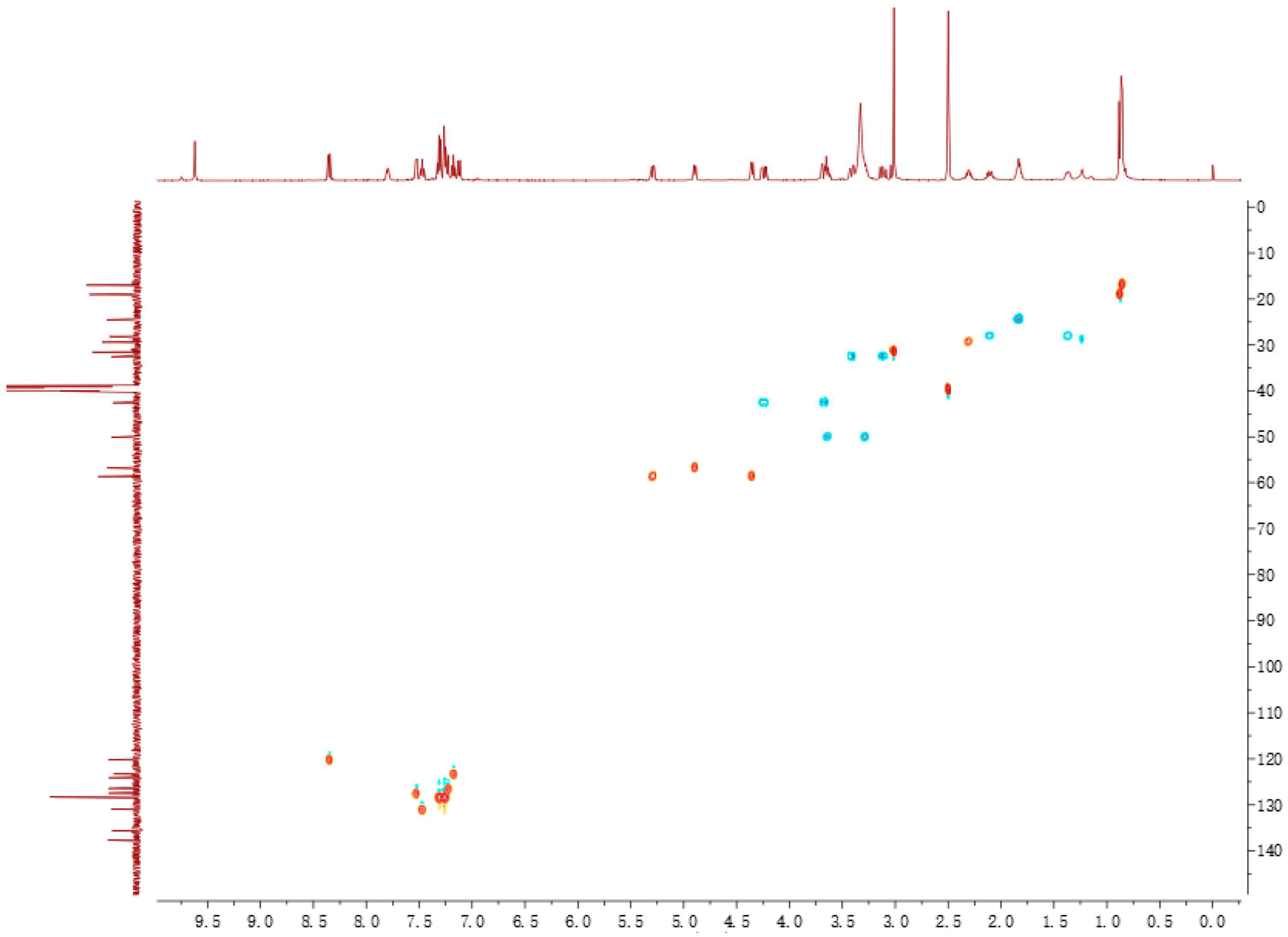

Figure S27. HMQC spectrum of **3**.

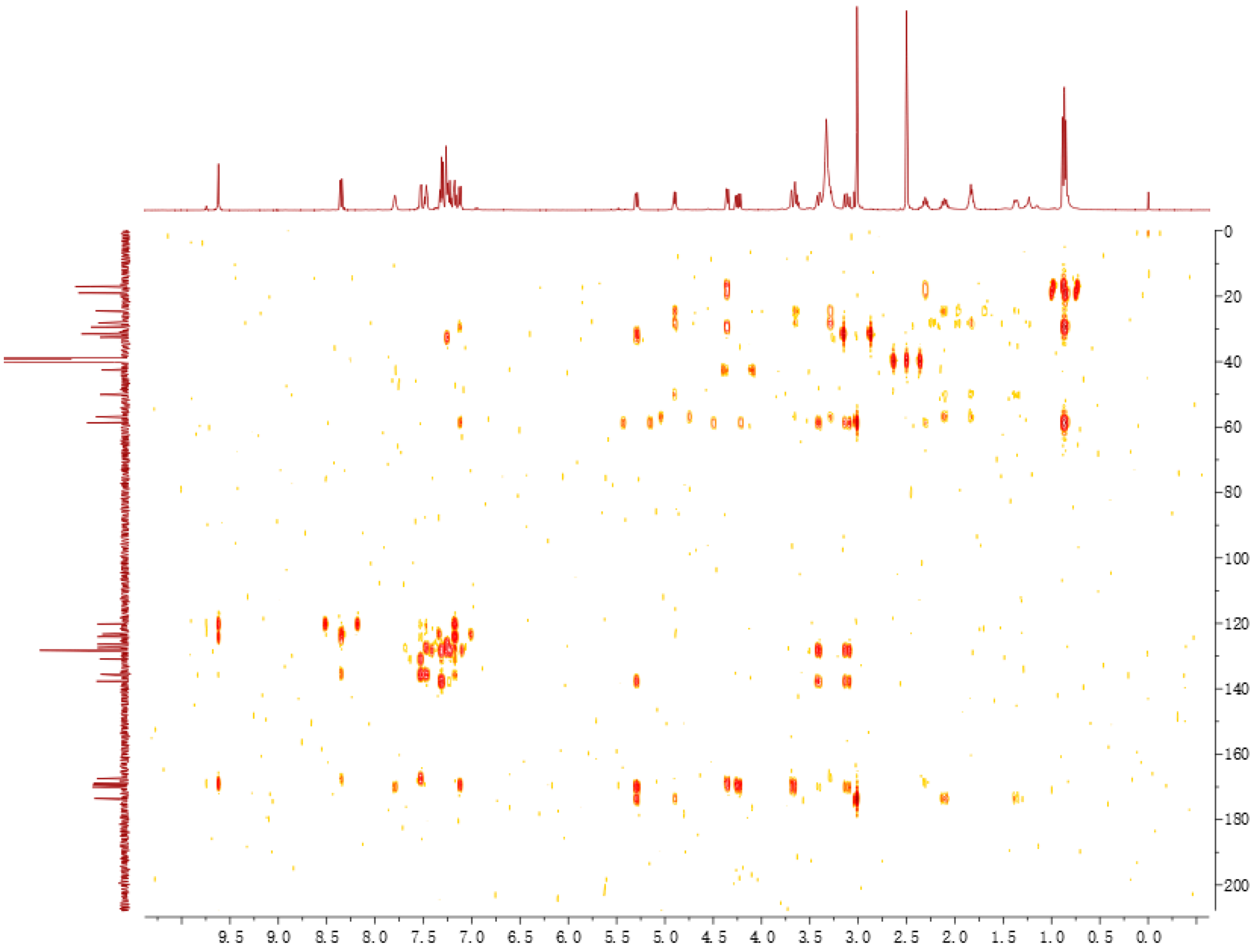

Figure S28. HMBC spectrum of **3**.

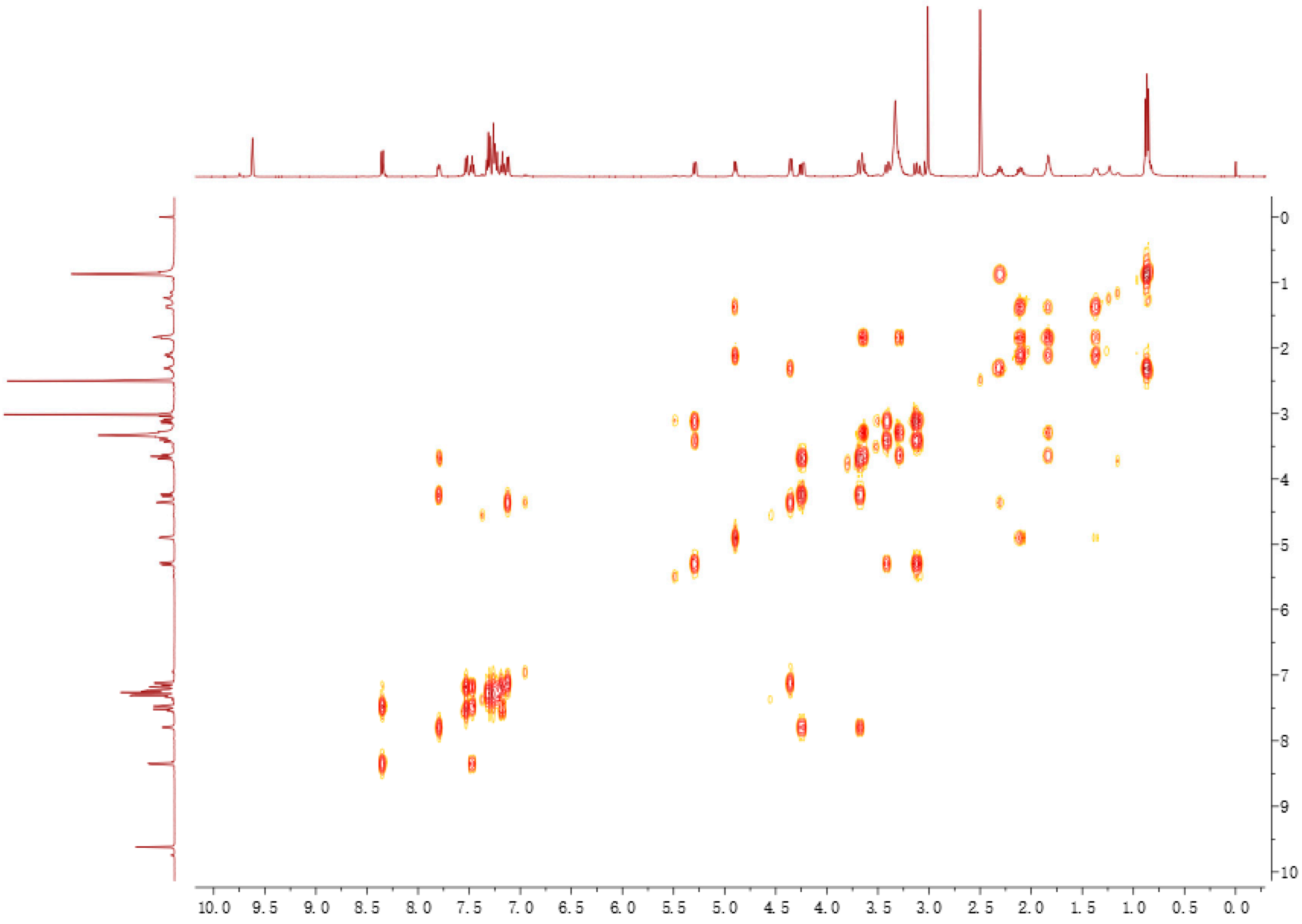

Figure S29. ^{1}H-^{1}H COSY spectrum of **3**.

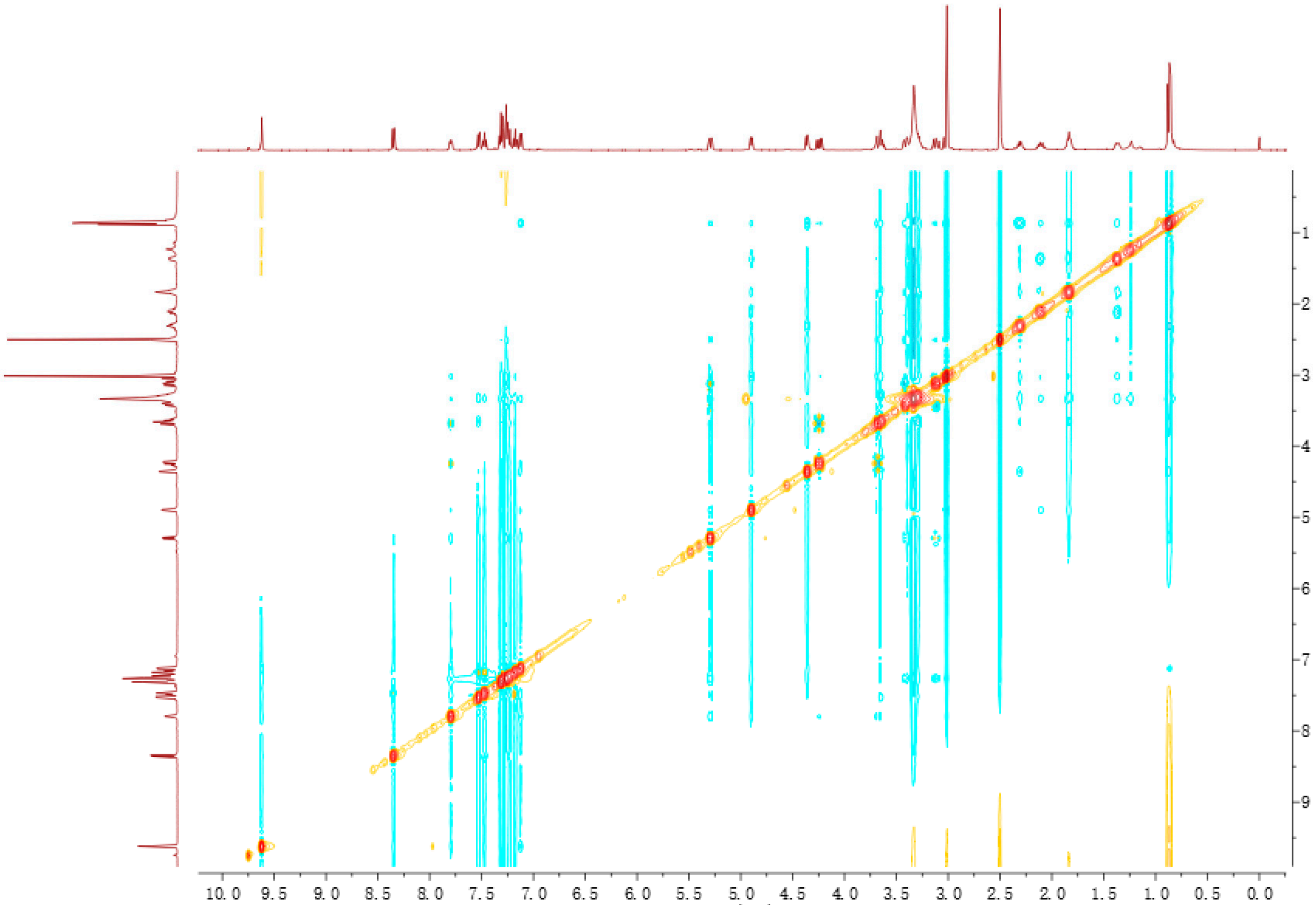

Figure S30. NOESY spectrum of **3**.

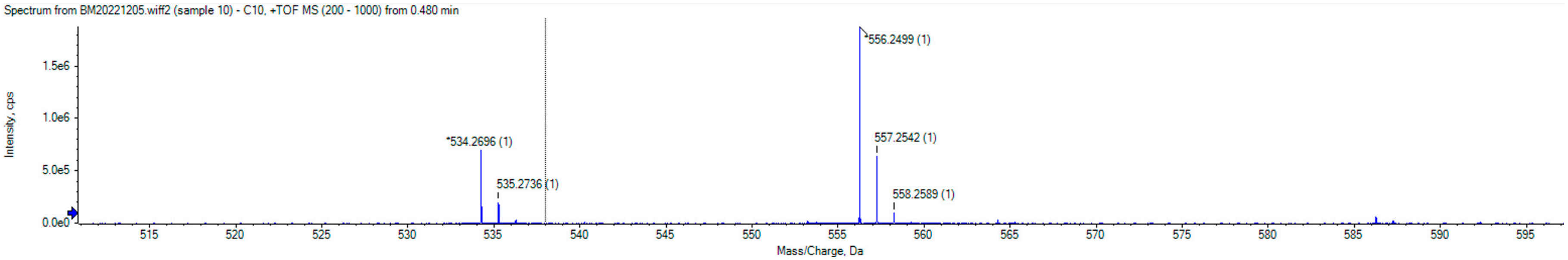

Figure S31. HR-ESI-MS spectrum of **3**.

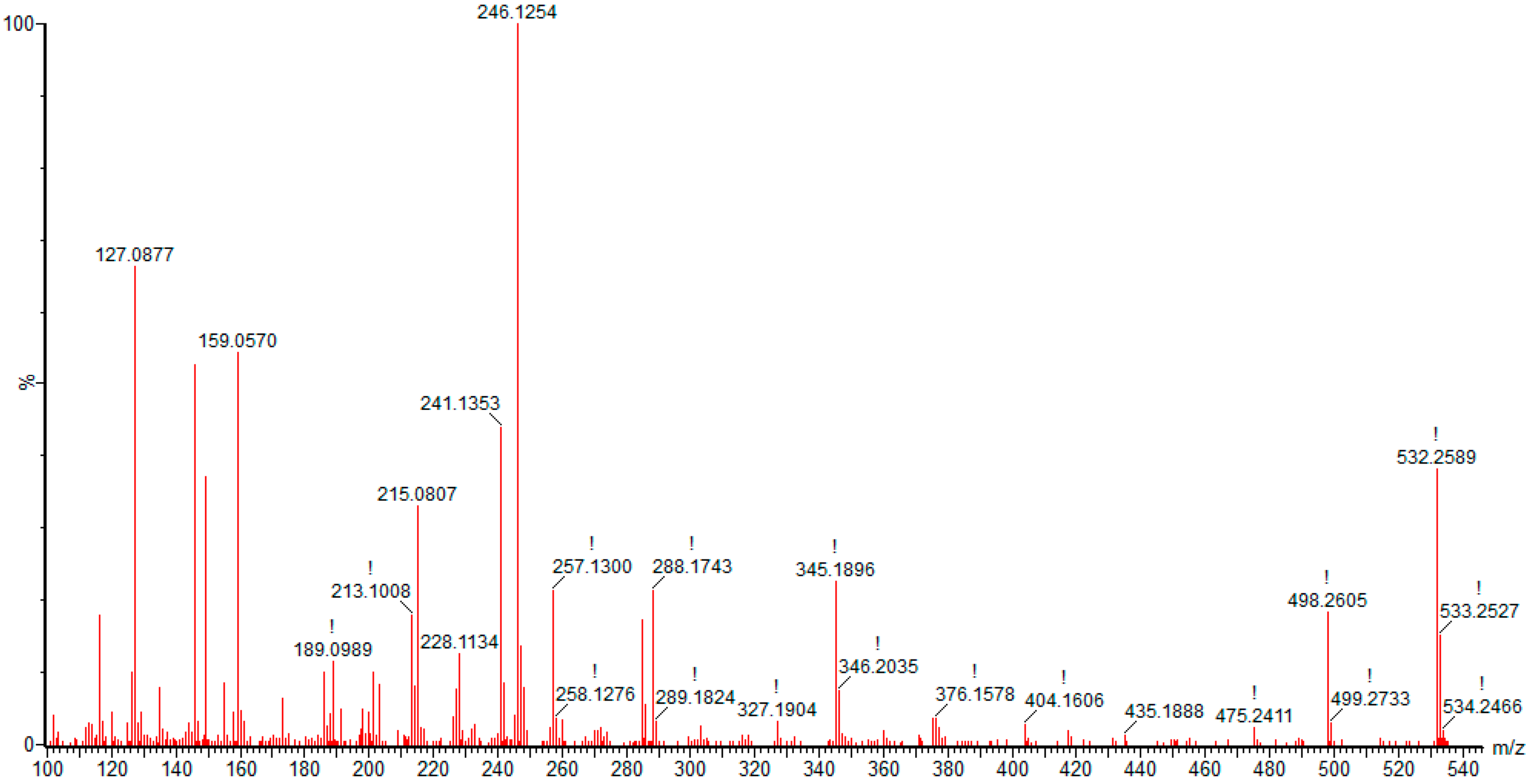

Figure S32. ESI-MS/MS spectrum of **3**.

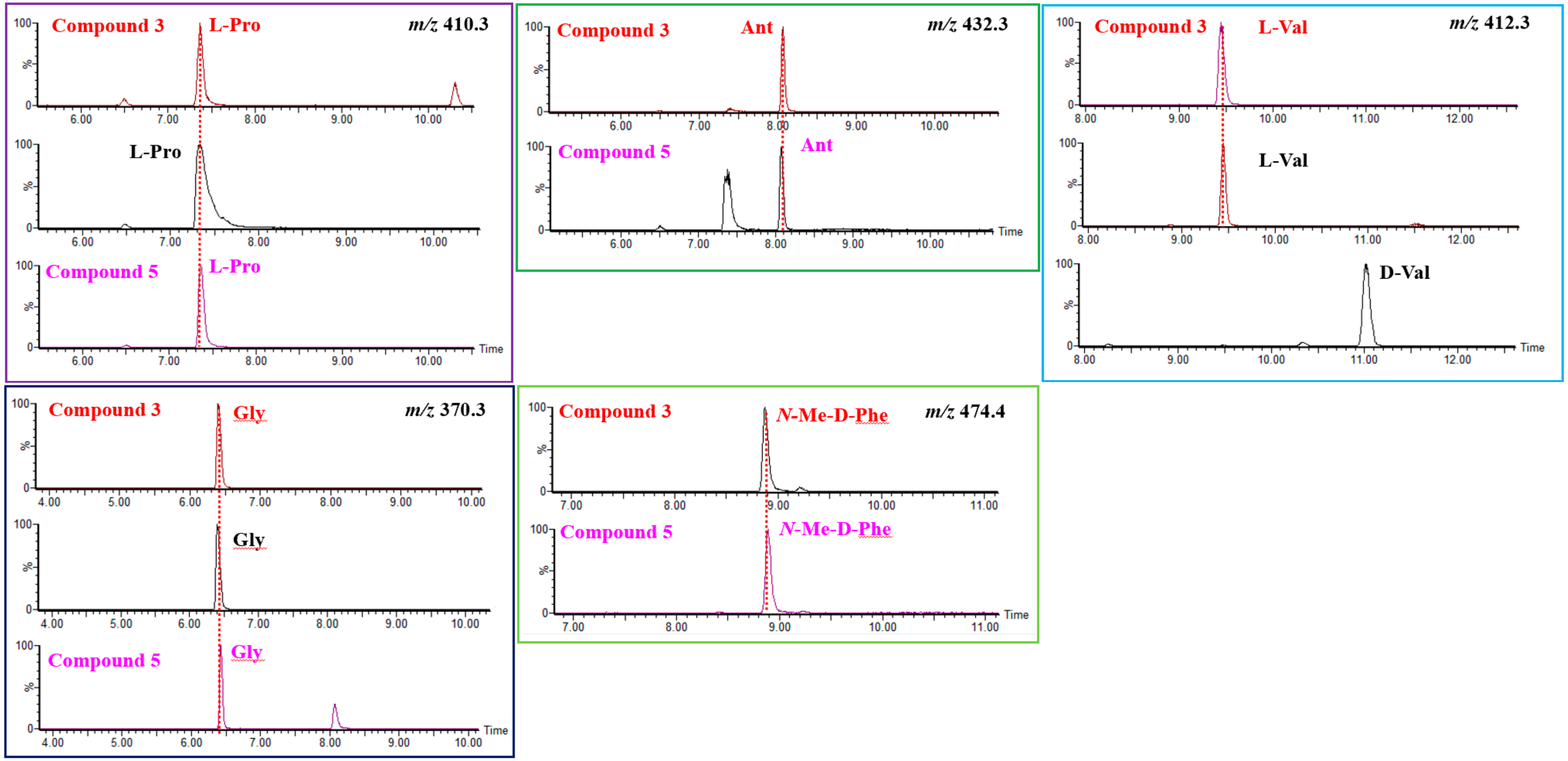

Figure S33. Marfey's analysis of **3**.

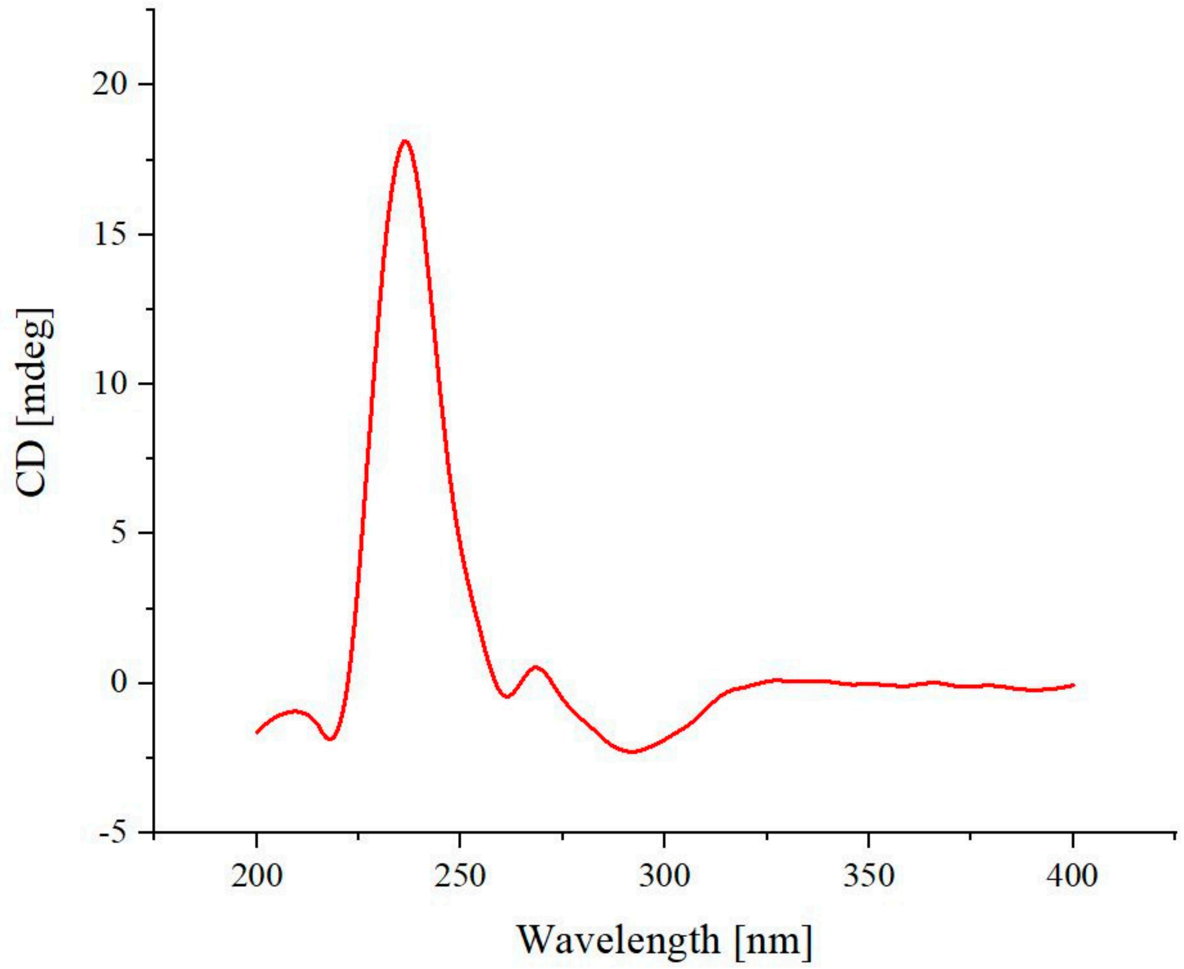

Figure S34. CD spectrum of **3** in MeOH.

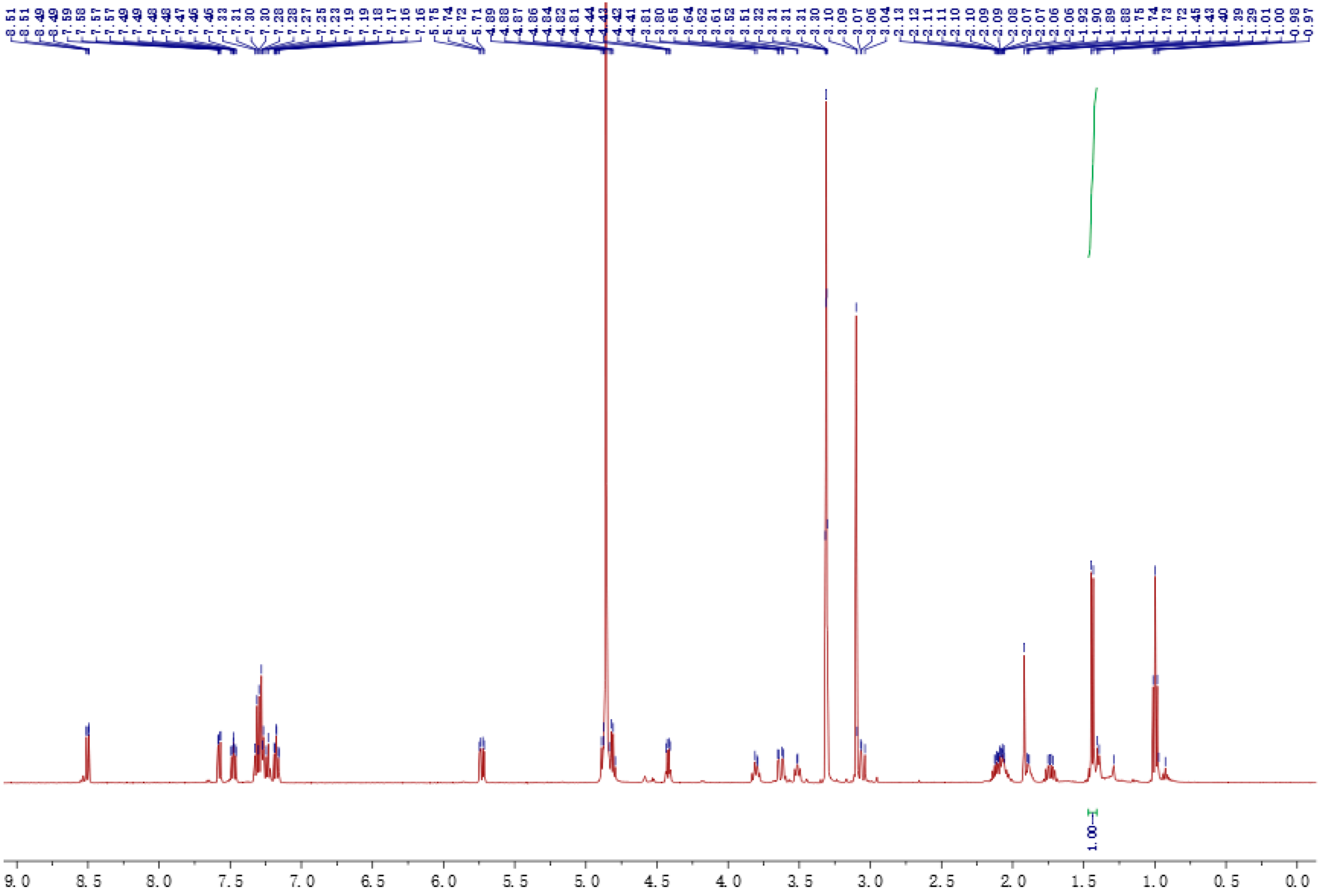

Figure S35. ^{1}H NMR (CD_3OD, 500 MHz) spectrum of **4**.

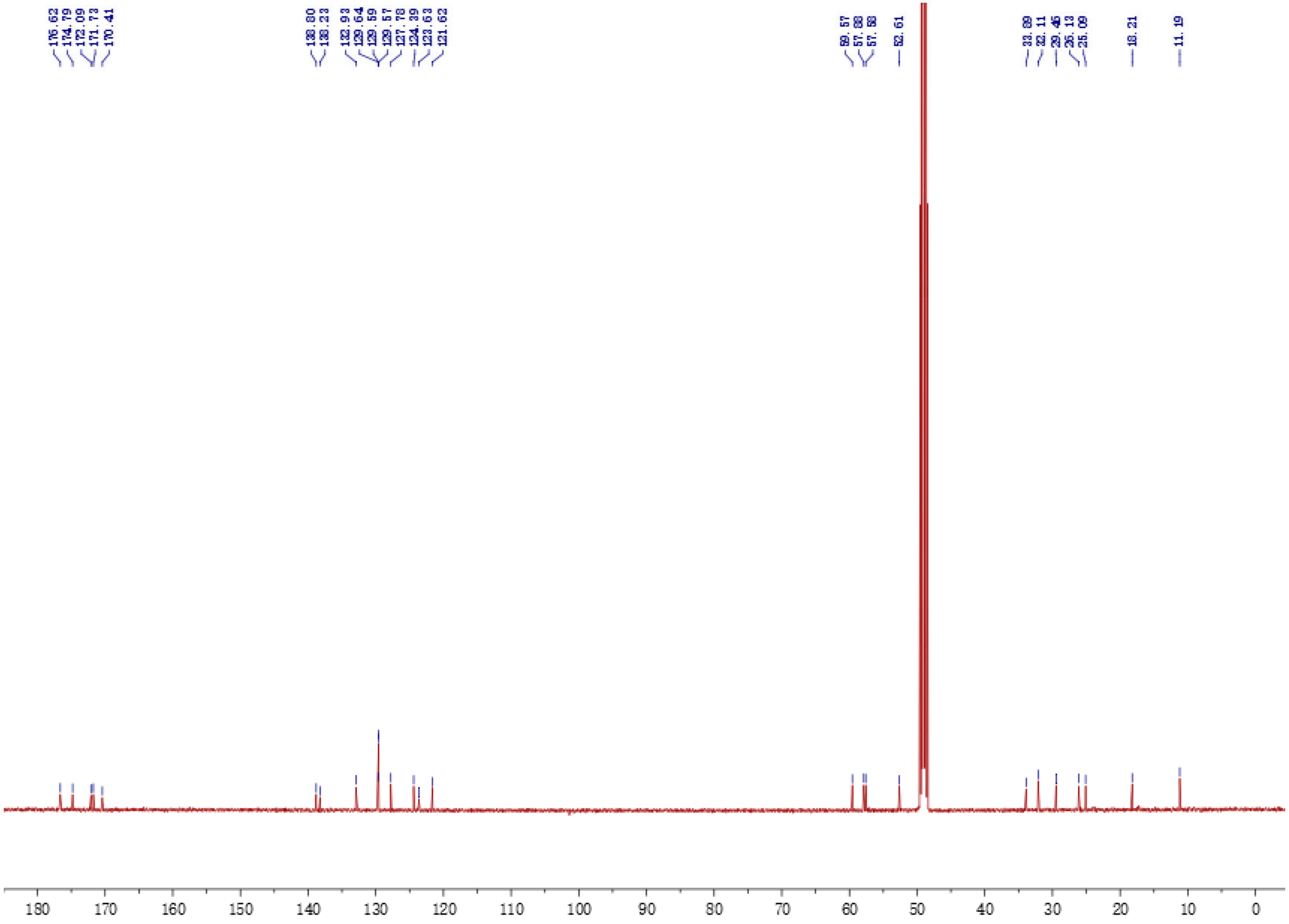

Figure S36. ^{13}C NMR (CD_3OD, 125 MHz) spectrum of **4**.

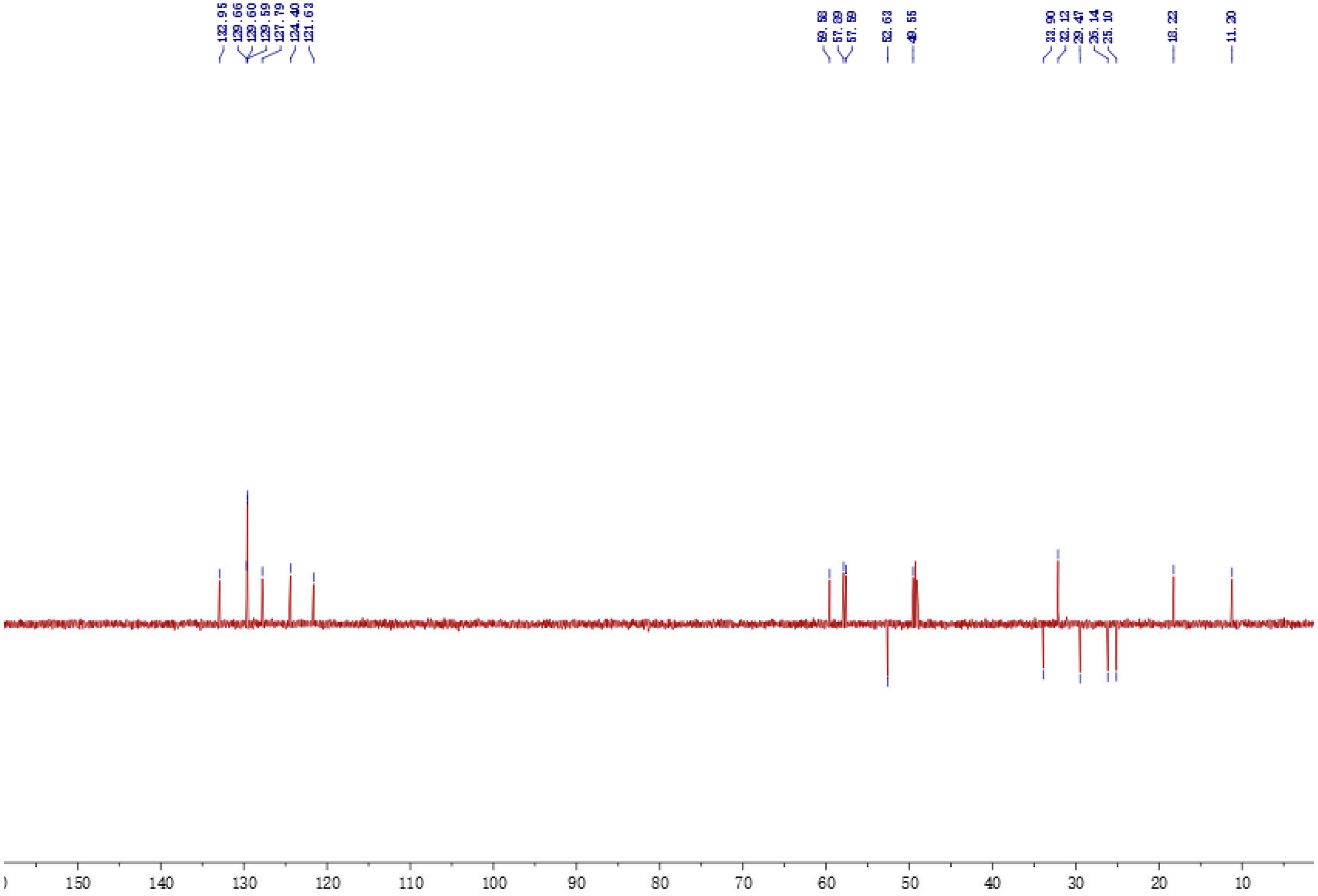

Figure S37. DEPT (CD_3OD, 125 MHz) spectrum of **4**.

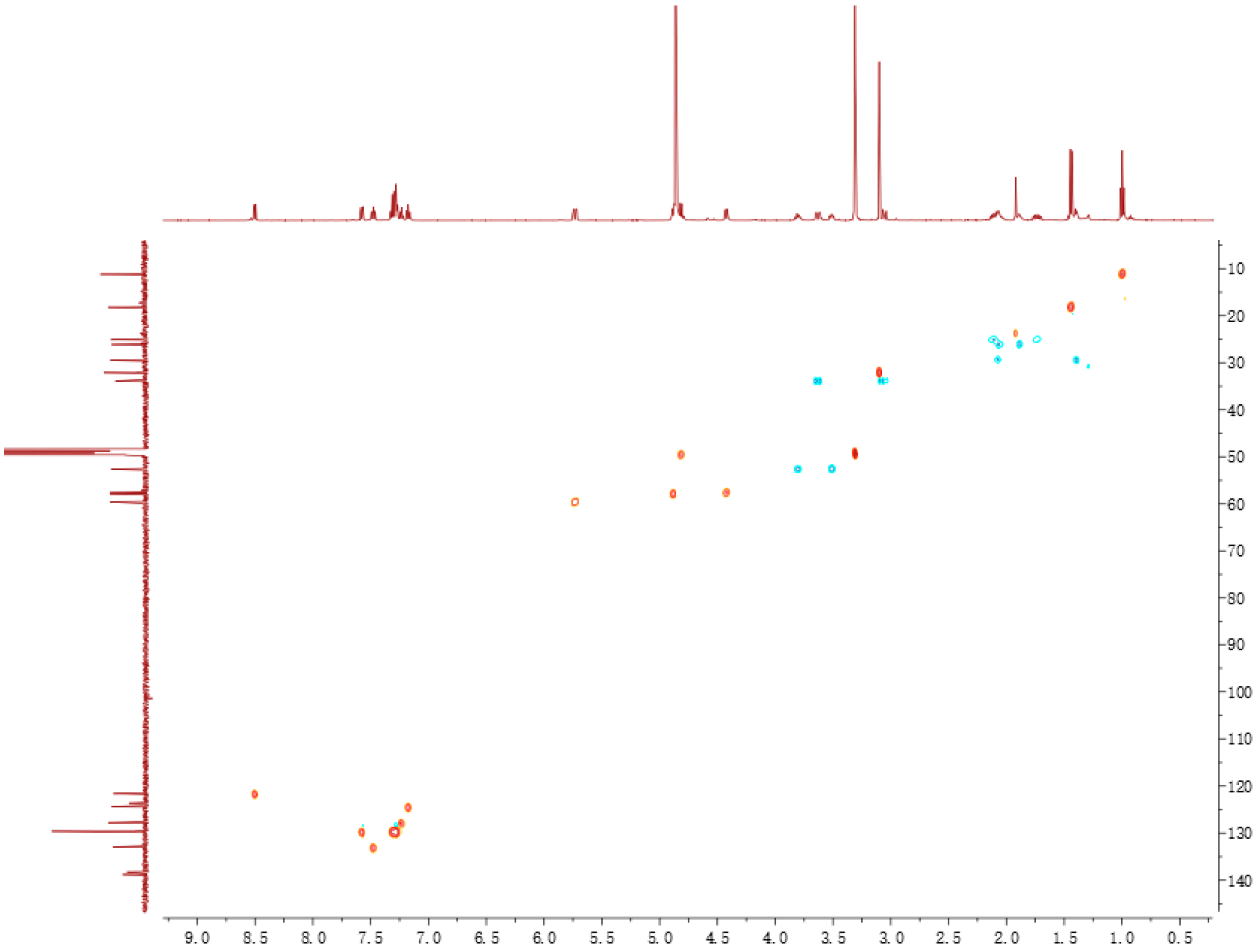

Figure S38. HMQC spectrum of **4**.

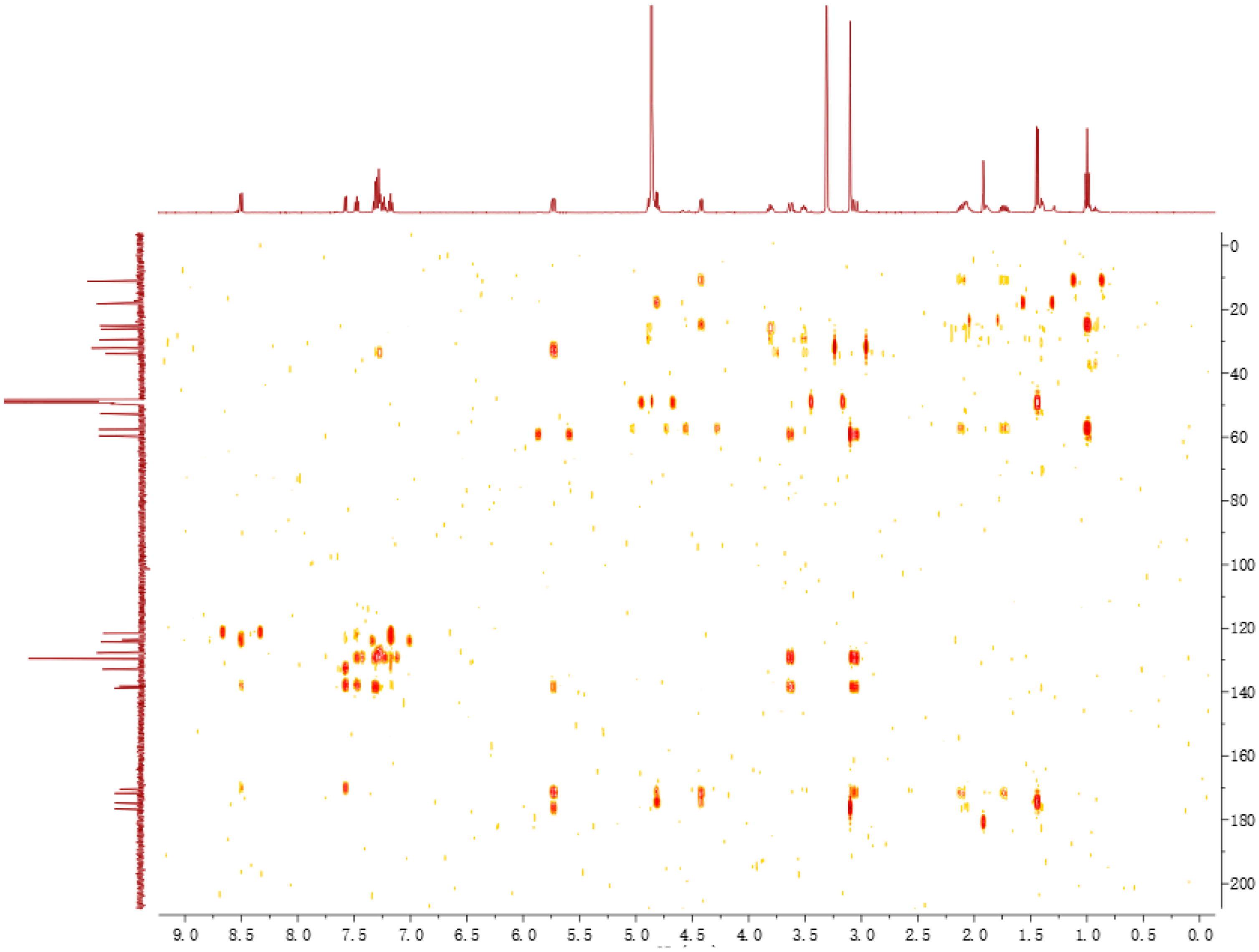

Figure S39. HMBC spectrum of **4**.

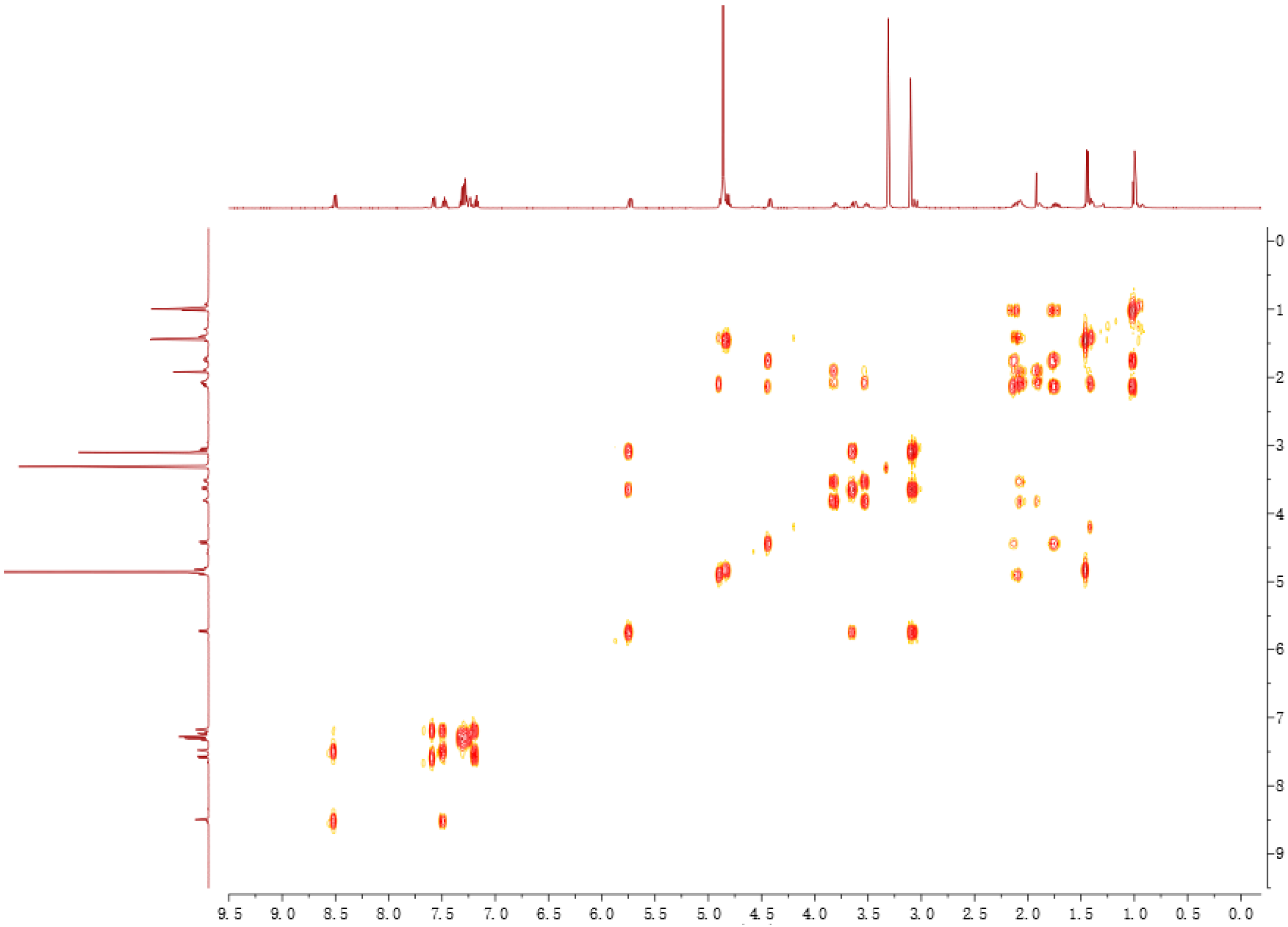

Figure S40. ^1H-^1H COSY spectrum of **4**.

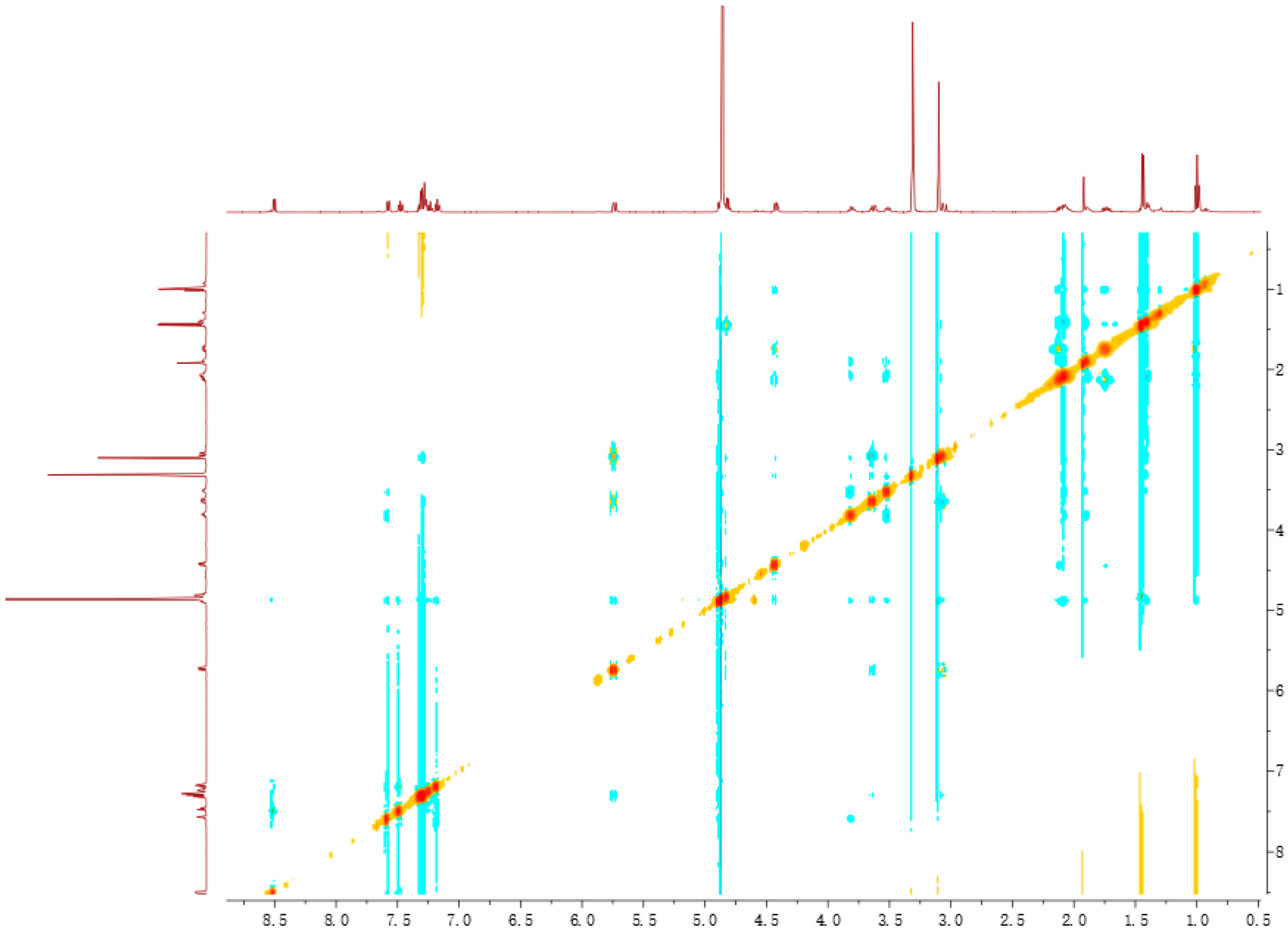

Figure S41. NOESY spectrum of **4**.

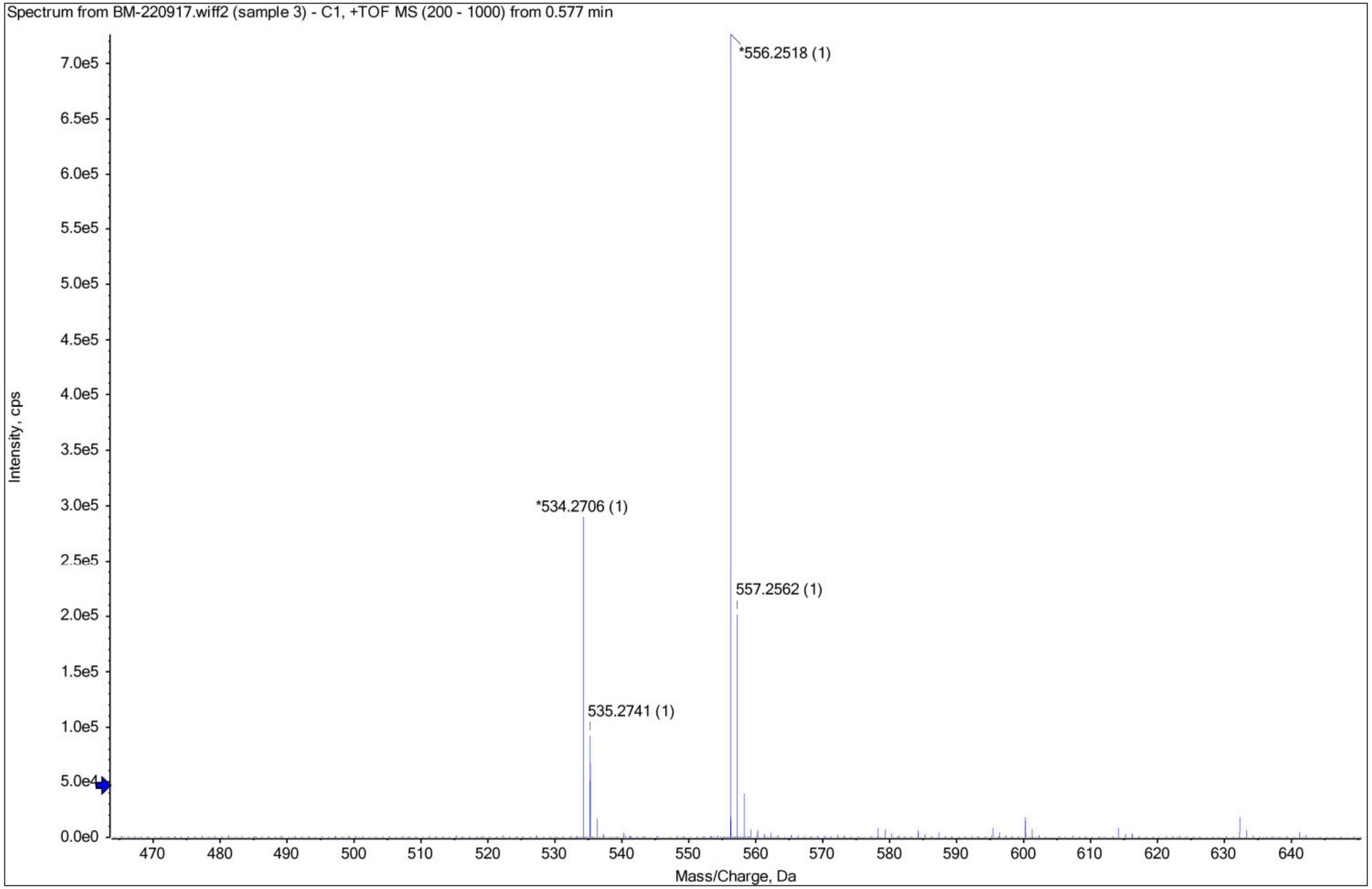

Figure S42. HR-ESI-MS spectrum of **4**.

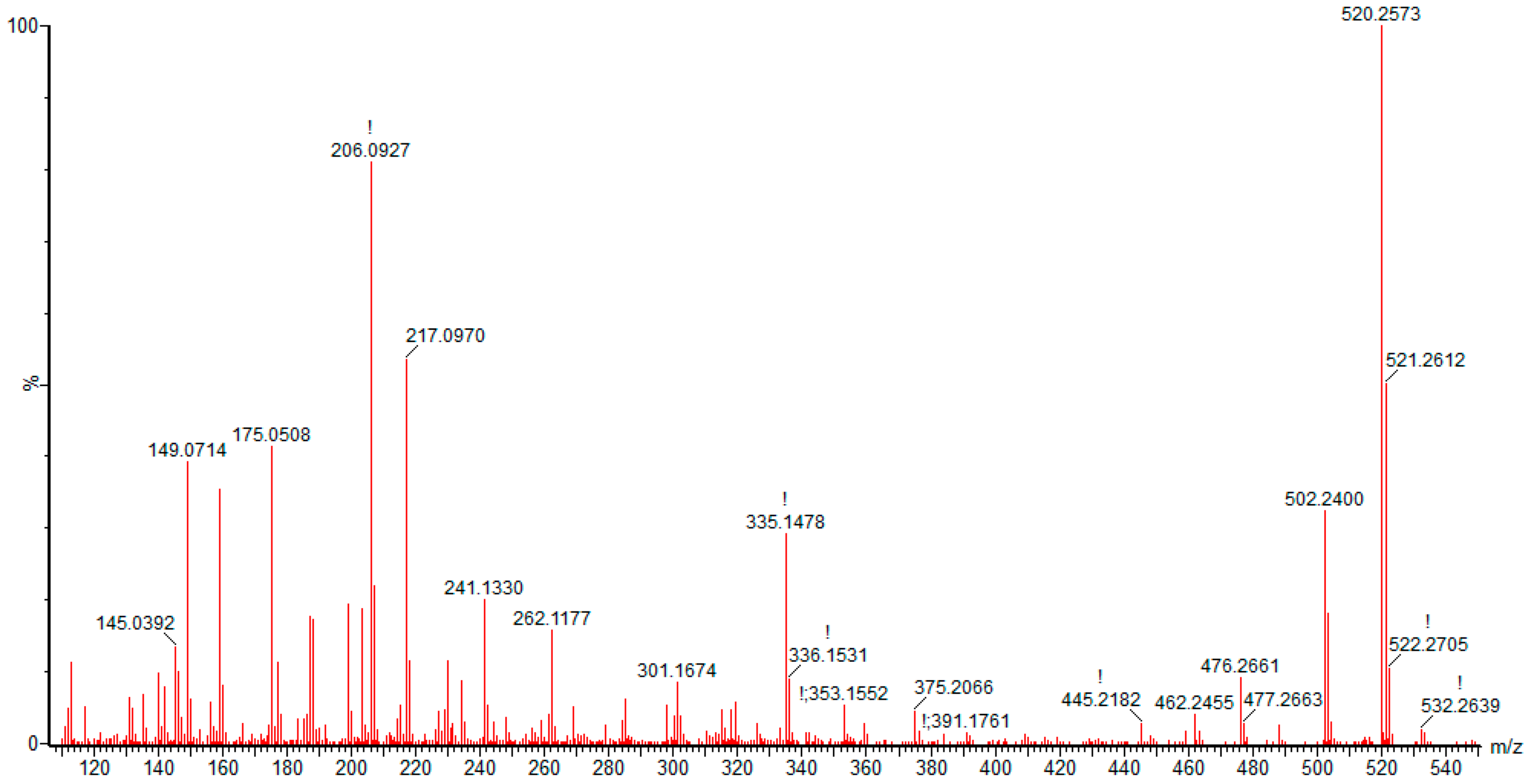

Figure S43. ESI-MS/MS spectrum of **4**.

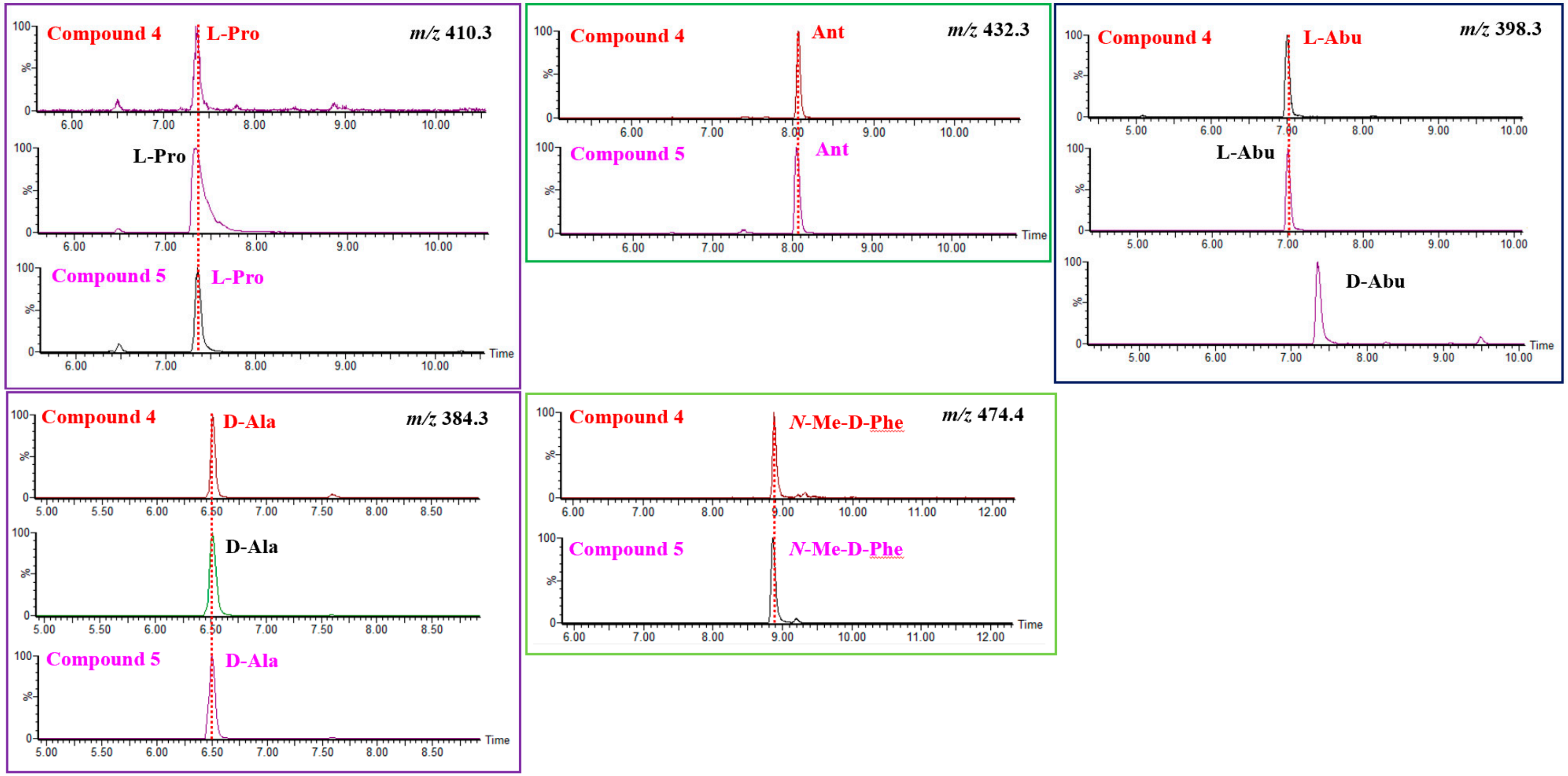

Figure S44. Marfey's analysis of **4**.

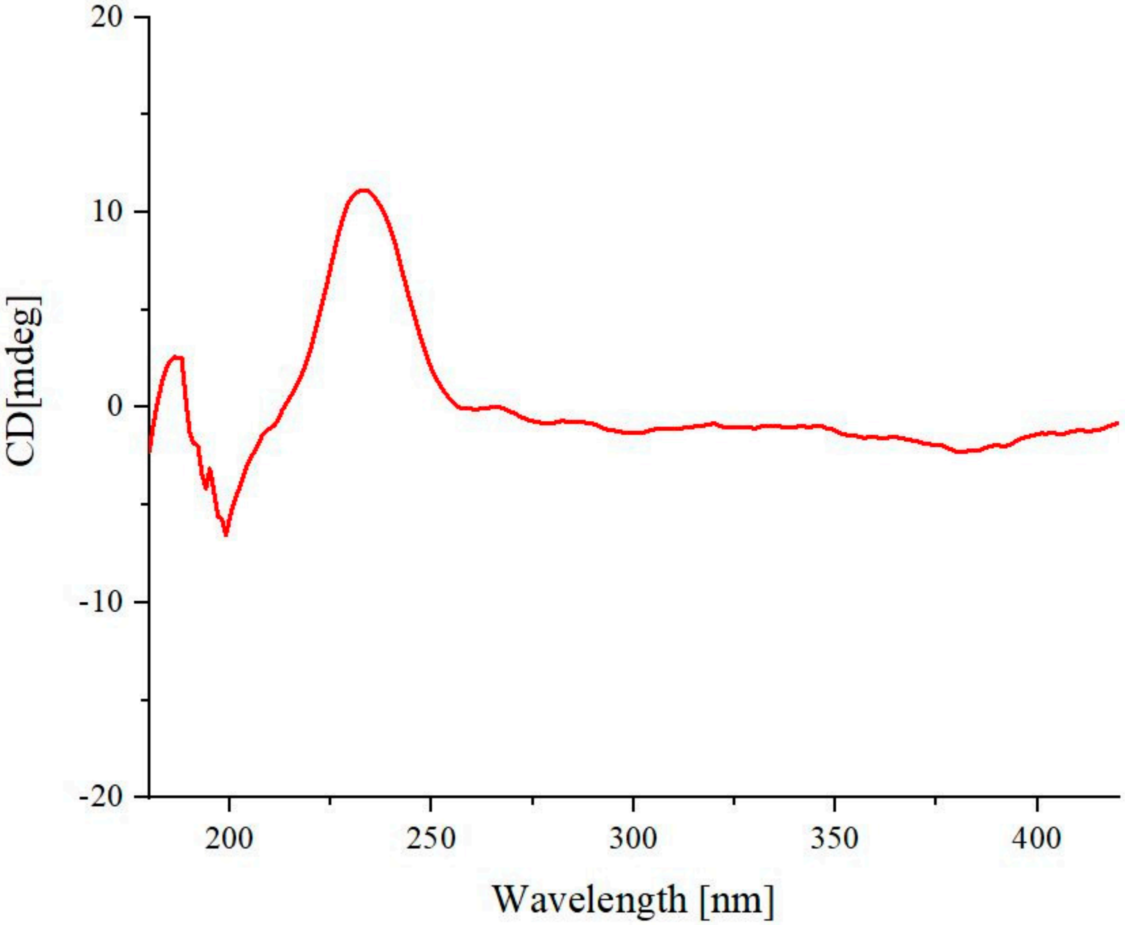

Figure S45. CD spectrum of **4** in MeOH.

Crystal data and structure refinement for **5**

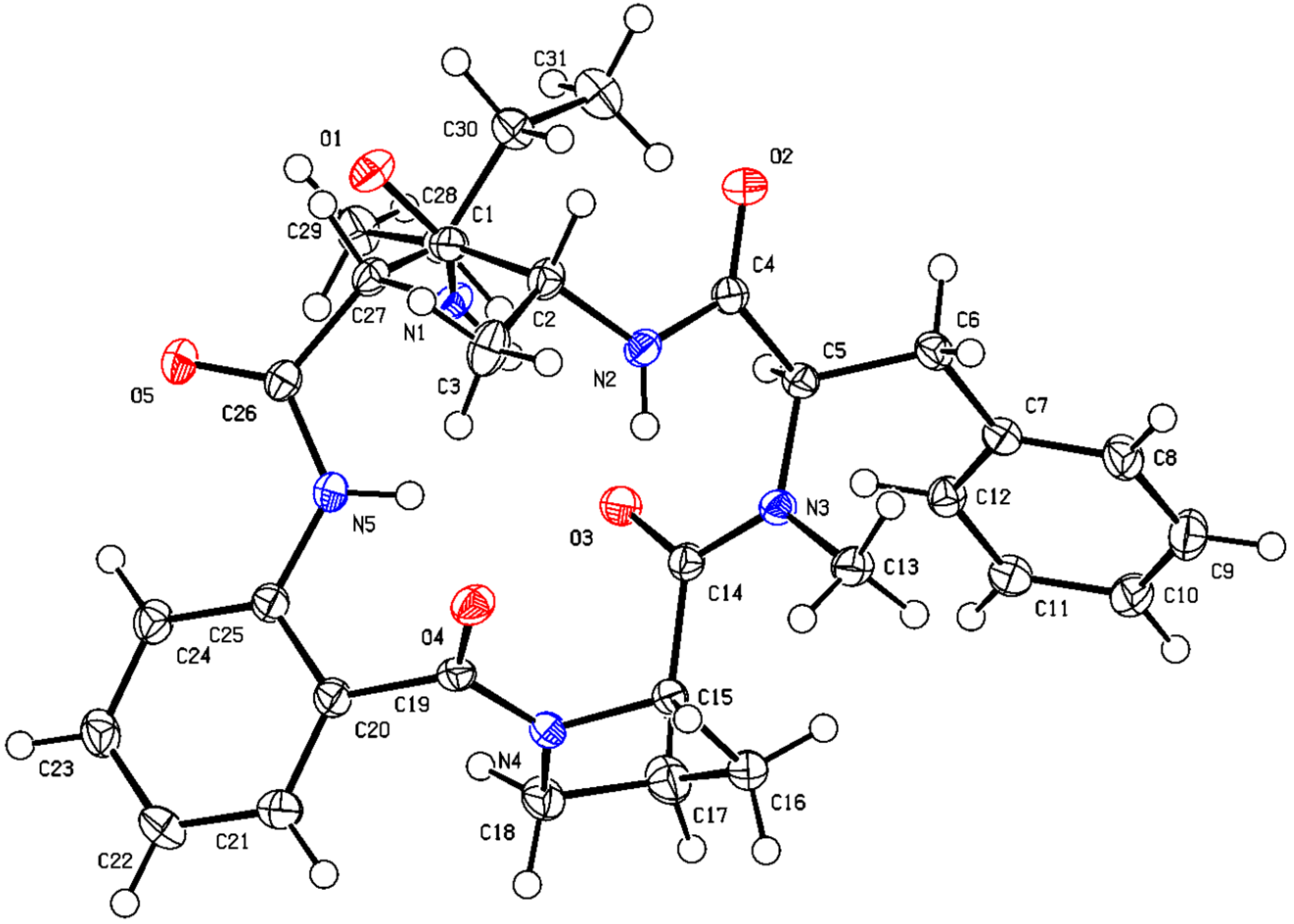

Empirical formula	$C_{31}H_{39}N_5O_5$
Formula weight	561.67
Temperature/K	99.9(3)
Crystal system	monoclinic
Space group	$P2_1$
a/Å	7.82520(10)
b/Å	18.63970(10)
c/Å	10.36950(10)
α/°	90
β/°	107.4970(10)
γ/°	90
Volume/Å^3	1442.51(3)
Z	2
ρ_{calc}g/cm^3	1.293
μ/mm^{-1}	0.721
F(000)	600.0
Crystal size/mm^3	0.3 × 0.25 × 0.2
Radiation	Cu Kα (λ = 1.54184)
2Θ range for data collection/°	8.942 to 148.786
Index ranges	$-9 \le h \le 9$, $-22 \le k \le 23$, $-11 \le l \le 12$
Reflections collected	26446
Independent reflections	5709 [R_{int} = 0.0242, R_{sigma} = 0.0132]
Data/restraints/parameters	5709/1/375
Goodness-of-fit on F^2	1.074
Final R indexes [I>=2σ (I)]	R_1 = 0.0274, wR_2 = 0.0713
Final R indexes [all data]	R_1 = 0.0274, wR_2 = 0.0714
Largest diff. peak/hole / e Å^{-3}	0.16/-0.24
Flack parameter	0.06(6)
